# Proteome and metabolome profiling of cytokinin action in *Arabidopsis* identifying both distinct and similar responses to cytokinin down- and up-regulation

**DOI:** 10.1093/jxb/ert227

**Published:** 2013-09-24

**Authors:** Martin Černý, Alena Kuklová, Wolfgang Hoehenwarter, Lena Fragner, Ondřej Novák, Gabriela Rotková, Petr L. Jedelský, Kateřina Žáková, Mária Šmehilová, Miroslav Strnad, Wolfram Weckwerth, Břetislav Brzobohatý

**Affiliations:** ^1^Laboratory of Plant Molecular Biology, Institute of Biophysics AS CR and CEITEC–Central European Institute of Technology, Mendel University in Brno, Zemědělská 1, CZ-613 00 Brno, Czech Republic; ^2^Department of Molecular Systems Biology (MOSYS), University of Vienna, Althanstrasse 14, A-1090 Vienna, Austria; ^3^Laboratory of Growth Regulators, Palacký University and Institute of Experimental Botany, Academy of Sciences of the Czech Republic, CZ-78371 Olomouc, Czech Republic; ^4^Department of Cell Biology, Faculty of Science, Charles University, Viničná 7, CZ-128 43 Prague, Czech Republic; ^5^Centre of the Region Haná for Biotechnological and Agricultural Research, Department of Molecular Biology, Faculty of Science, Palacký University, Šlechtitelů 11, CZ-78371 Olomouc, Czech Republic

**Keywords:** *Arabidopsis thaliana*, cytokinin, cytokinin oxidase/dehydrogenase, isopentenyl transferase, metabolome, proteome.

## Abstract

In plants, numerous developmental processes are controlled by cytokinin (CK) levels and their ratios to levels of other hormones. While molecular mechanisms underlying the regulatory roles of CKs have been intensely researched, proteomic and metabolomic responses to CK deficiency are unknown. Transgenic *Arabidopsis* seedlings carrying inducible barley cytokinin oxidase/dehydrogenase (*CaMV35S*>GR>*HvCKX2*) and agrobacterial isopentenyl transferase (*CaMV35S*>GR>*ipt*) constructs were profiled to elucidate proteome- and metabolome-wide responses to down- and up-regulation of CK levels, respectively. Proteome profiling identified >1100 proteins, 155 of which responded to *HvCKX2* and/or *ipt* activation, mostly involved in growth, development, and/or hormone and light signalling. The metabolome profiling covered 79 metabolites, 33 of which responded to *HvCKX2* and/or *ipt* activation, mostly amino acids, carbohydrates, and organic acids. Comparison of the data sets obtained from activated *CaMV35S*>GR>*HvCKX2* and *CaMV35S*>GR>*ipt* plants revealed unexpectedly extensive overlaps. Integration of the proteomic and metabolomic data sets revealed: (i) novel components of molecular circuits involved in CK action (e.g. ribosomal proteins); (ii) previously unrecognized links to redox regulation and stress hormone signalling networks; and (iii) CK content markers. The striking overlaps in profiles observed in CK-deficient and CK-overproducing seedlings might explain surprising previously reported similarities between plants with down- and up-regulated CK levels.

## Introduction

Cytokinins (CKs) regulate diverse developmental processes in plants, including: the formation and activity of shoot meristems; apical dominance; leaf senescence; nutrient mobilization; seed germination; root, flower, and fruit development; plant–pathogen interactions; and stress responses. They also participate in various light-regulated processes, such as de-etiolation and chloroplast differentiation (e.g. Lochmanová *et al.*, 2008; Argueso *et al.*, 2009; Perlilli *et al.*, 2010; Brenner *et al.*, 2012; Novák *et al.*, 2013).

Naturally occurring CKs are adenine derivatives substituted at the *N* ^6^-position with an isoprenoid or aromatic side chain. CK metabolism has been extensively reviewed (e.g. Strnad, 1997; Sakakibara, 2006; Kudo *et al.*, 2010; Frébort *et al.*, 2011). Briefly, the first step in isoprenoid CK biosynthesis, formation of isopentenyladenosine-5′-phosphates (iP-nucleotides) by transfer of an isopentenyl group to adenosine-5′-phosphates, is catalysed by adenosine phosphate-isopentenyl transferase (IPT; EC 2.5.1.27). The iP-nucleotides can be transformed to the corresponding zeatin-nucleotides, which are subsequently converted to active (free-base) CKs. CKs are inactivated either by conjugation, mainly reversible *O*-glucosylation and irreversible *N* ^7^- or *N* ^9^-glucosylation, or by degradation.

A key enzyme in CK inactivation is cytokinin oxidase/dehydrogenase (CKX; EC 1.5.99.12), which degrades CK bases, ribosides, nucleotides, and *N* ^9^ *-*glucosides by oxidative cleavage of the *N* ^6^ *-*unsaturated CK side chain, resulting in the formation of a side chain-derived aldehyde and adenine (or corresponding derivatives) (Galuszka *et al*., 2001, 2007; Kowalska *et al.*, 2010). CKX activity was first found in crude tobacco culture in 1971 (Pačes *et al.*, 1971) and it remains the only characterized enzymatic CK degradation mechanism in plants. Generally, only *N* ^*7*^-glucosides, *O*-glucosides, and dihydrozeatins are not susceptible to CKX degradation.

Constitutive ectopic overexpression in transgenic plants of both IPTs (e.g. Smigocki and Owens, 1988; Cline, 1994) and CKXs (e.g. Werner *et al*., 2001, 2003, 2010; Bartrina *et al.*, 2011; Holst *et al.*, 2011; Nishiyama *et al.*, 2011) has dramatically contributed to knowledge of CK action. However, use of constitutive promoters has inherent limitations. For example, CKs are homeostastically regulated by a fine balance between synthesis, conjugation, and degradation. Synthesis was originally believed to occur in root apical meristems, but transcriptomic analysis has revealed that CKs are produced in discrete sites throughout the plant in diverse organs and tissues, including roots, shoots, and vascular tissues, at specific developmental stages (Sakakibara, 2006; Argueso *et al.*, 2009; Perilli *et al.*, 2010). CK receptors are also widely distributed both spatially and temporally in plants (Stolz *et al.*, 2011). Thus, the constitutive overexpression of CK metabolism enzymes is clearly problematic for studying the fine tuning of CK signalling or elucidating the roles of CKs at different developmental stages. However, several systems are available for chemically inducing transgene expression in plants, thus offering possibilities for restricting transgene expression to a particular developmental stage or generation (e.g. Moore *et al.*, 2006). One of these, a stringently regulated and highly responsive dexamethasone (DEX)-inducible gene expression system, has been used to study the effects of activating an agrobacterial isopentenyl transferase gene, *ipt*, in *Arabidopsis* (Craft *et al.*, 2005; Hradilová *et al.*, 2007; Lochmanová *et al.*, 2008) and tobacco (Šámalová *et al.*, 2005).

The molecular mechanisms underlying CK action have been intensively researched, but not the proteome- and metabolome-wide changes triggered by controlled reductions in bulk CK levels. Therefore, dynamic global responses in *Arabidopsis* seedlings to reductions and increases in the bulk CK pool, mediated by DEX-induced activation of a construct hosting *HvCKX2*, a barley CKX isoform 2 gene (Galuszka *et al.*, 2004), have been examined. The proteomic and metabolomic responses to inducible *HvCKX2* and *ipt* activation have also been compared. Here the results of these analyses are presented, and the implications of significant overlaps found in the responses, notably indications that homeostatic control mechanisms with opposite effects are triggered by CK depletion and overproduction, are discussed.

## Materials and methods

### Plant material, growth conditions, and dexamethasone treatment

The plants used in the experiments were transgenic *CaMV35S*>GR>*HvCKX2* line 13, *CaMV35S*>GR>*ipt* line pOp^BK^-ipt 11 (Craft *et al.*, 2005), and corresponding wild-type plants (*Arabidopsis thaliana* ecotype Col-0). Briefly, DEX-inducible *CaMV35S*>GR>*HvCKX2* lines were prepared as follows. The genomic sequence of barley HvCKX2 (AF490591) was subcloned from the pDRIVE vector (Galuszka *et al.*, 2004) into Gateway entry pENTR1A vector *Kpn*I/*Not*I sites (Invitrogen) with *Acc*65I/*Not*I restriction enzymes. Subsequently, the gene was inserted into the pOpOn2.1 binary vector derived from the pOpOff2 vector (Wielkopolska *et al*., 2005; provided by Dr Ian Moore, Department of Plant Sciences, University of Oxford) downstream of the artificial pOp6 promoter (Supplementary Fig. S1 available at *JXB* online) via a Gateway LR clonase II recombination reaction (Invitrogen). *Arabidopsis thaliana* ecotype Columbia Col-0 plants were transformed by the flower-dip procedure (Bechtold *et al.*, 1993). Based on screening for the DEX-induced cytokinin deficiency phenotype, a strongly responsive line 13 was selected out of 20 independent transformants and selfed to obtain homozygous offspring. A seed stock for large-scale experiments was harvested from T_3_ plants. Seeds were surface-sterilized and sown on Uhelon 120T (Silk & Progress, www.silkandprogress.cz) mesh placed on 1% (w/v) agar containing Murashige and Skoog (MS) medium (pH 5.7) supplemented with 5×10^–4^% (v/v) dimethylsulphoxide (DMSO), stratified at 4 °C for 3 d, and cultivated at 21 °C/19 °C day/night temperatures, with a 16h photoperiod (80 µmol m^–2^ s^–1^ light intensity) for 7 d in a growth chamber (AR36LX, Percival, http://www.percival-scientific.com/). Prior to the harvest, the Uhelon mesh with the seedlings was transferred onto fresh MS medium supplemented with 5×10^–4^% (v/v) DMSO (mock) or 20 µM DEX (Sigma-Aldrich, http://www.sigmaaldrich.com) in DMSO (final concentration, as for the mock) for: (i) 6, 12, 24, and 48h for analysis of endogenous CK levels following DEX induction; (ii) 12h and 48h for two-dimensional electrophoresis (2-DE) proteomic analysis; (iii) 48h for liquid chromatography–mass spectrometry (LC-MS) proteomic and metabolomic analysis, and quantitative reverse transcription–PCR (RT–qPCR) analysis. Seedlings were then harvested and frozen in liquid nitrogen.

### 2-DE MALDI TOF/TOF proteome profiling

Protein was extracted from frozen seedlings (180mg, ~250 seedlings), loaded onto Seppro IgY-Rubisco Spin Columns (Sigma-Aldrich), and processed according to the supplier’s manual. RuBisCO-depleted samples were pooled and extracted by acetone/trichloroacetric acid (TCA) extraction (Damerval *et al.*, 1986). Dried protein was solubilized and separated, essentially as previously described (Lochmanová *et al.*, 2008; Hradilová *et al.*, 2010; Černý *et al.*, 2011*a*, *b*). Briefly, portions of extracts containing 150 µg of protein were applied to 7cm IPG strips with a non-linear pH gradient (3–10; Bio-Rad, http://www.bio-rad.com/), isoelectrically focused, then treated with buffers containing dithiothreitol (DTT) and iodoacetamide (Sigma-Aldrich) to reduce and alkylate the proteins, which were subsequently separated by SDS–PAGE. Gels were stained with colloidal Bio-Safe Coomassie G-250 (Bio-Rad), digitally imaged, and analysed using Decodon Delta 2D software (http://www.decodon.com). Two biological replicates with three technical replicates were used in the comparisons. Responses to DEX activation of proteins corresponding to detected spots were deemed significant if there was an absolute DEX/mock spot volume ratio ≥1.4, with *t*-test *P*-values <0.05 and similar profiles in two biological replicates. Spots with significant and reproducible changes were cut, digested with trypsin (Promega, http://www.promega.com/), desalted, and the resulting protein fragments were analysed using a 4800 Plus matrix-assisted laser desorption tandem time of flight (MALDI TOF/TOF) instrument (AB Sciex, http://www.absciex.com/). For details, see Supplementary Methods S1 at *JXB* online.

### LC-MS proteome profiling

Further proteomic analyses were performed using a gel-free shotgun protocol based on nano-high-perfomance liquid chromatography (HPLC) and tandem mass spectrometry (MS/MS), as described elsewhere (e.g. Larrainzar *et al.*, 2007). Briefly, two biological replicates, each consisting of ~300 *Arabidopsis* seedlings cultivated as described above, were pooled and analysed in three technical replicates. Proteins were extracted by combination of acetone/TCA and phenol extraction, and digested in solution with endoproteinase Lys-C and immobilized trypsin beads (Promega). The resulting peptides were desalted, dried, and dissolved in 0.5% (v/v) formic acid in 5% (v/v) acetonitrile, then analysed online by nanoflow C18 reverse-phase LC using a 15cm Ascentis Express Column (0.1mm inner diameter; Sigma-Aldrich) and an Eksigent ultra-high-performance liquid chromatography (UPLC) system (Eksigent, http://www.eksigent.com/) directly coupled to an electrospray ionization (ESI) source and an LTQ-Orbitrap XL mass spectrometer (Thermo Scientific, http://www.thermoscientific.com). Peptides were eluted with a 155min, 5–95% acetonitrile gradient. Dynamic exclusion settings were as described in Hoehenwarter and Wienkoop (2010). Raw files obtained from the MS analysis were searched against the TAIR10 *Arabidopsis* database using the Sequest algorithm. For identification and spectral count-based data matrix generation, Proteome Discoverer (v 1.3, Thermo Scientific) was used. Only high confidence peptides (false discovery rate <1%) with >7 ppm precursor mass accuracy and at least one distinct peptide per protein met identification criteria. Quantitative differences in protein abundance between DEX- and mock-treated samples were screened by spectral counting (Neilson *et al.*, 2011) and were further manually validated by comparing respective peptide ion signal peak areas (Qual Browser 2.0.7, Thermo Scientific). Quantitative differences were deemed significant if there was an absolute DEX/mock ratio ≥1.5, with *t*-test *P*-values <0.05. For details, see Supplementary Methods S1 at *JXB* online.

### GC-MS metabolome profiling

Polar metabolites were analysed essentially as described in Morgenthal *et al.* (2007). Briefly, two biological replicates, each consisting of ~100 *Arabidopsis* seedlings cultivated as described above, were pooled and analysed in three technical replicates. Metabolites were extracted with methanol/chloroform/distilled water [2.5:1:0.5 (v/v/v)], and clarified by centrifugation. The resulting polar phase was separated by adding 0.5ml of distilled water, dried, methoximated and silylated, then analysed using an Agilent 6890 gas chromatograph (Agilent, http://www.home.agilent.com) coupled to a Pegasus IV TOF mass spectrometer (LECO, http://www.leco.com/). The acquired data were analysed using ChromaTOF software (LECO). Quantitative differences in metabolite abundance were deemed significant if there was an absolute DEX/mock ratio ≥2.5, with *t*-test *P*-values <0.05. For details, see Supplementary Methods S1 at *JXB* online.

### Quantification and identification of endogenous cytokinins

Endogenous CK contents of duplicate samples were analysed using the method described by Novák *et al*. (2003), with modifications described by Novák *et al*. (2008). Briefly, 150–200mg fresh weight of *Arabidopsis* seedlings were extracted in Bieleski buffer (Bieleski, 1964). A cocktail of stable isotope-labelled CKs (each at 1 pmol per sample) was added to the extracts to check purification recovery, and samples were purified using a combination of cation (SCX-cartridge) and anion (DEAE-Sephadex-C18-cartridge) exchange, followed by immunoaffinity chromatography, using a wide range of immobilized CK-specific monoclonal antibodies. Three resulting fractions were then analysed by UPLC–electrospray tandem mass spectrometry (using an UPLC Acquity System linked to a Xevo TQ MS spectrometer, Waters, http://www.waters.com). The concentrations of the various CKs were calculated using a standard isotope dilution method (Rittenberg and Foster, 1940).

### RT–qPCR analysis

Total RNA was prepared from 50mg of seedlings that had been frozen in liquid nitrogen using TRIzol reagent (Invitrogen, http://www.invitrogen.com), and contaminating DNA was removed by DNase I. First-strand cDNA was prepared using SuperScript II reverse transcriptase (Invitrogen) and the oligo(dT) primer according to the manufacturer’s instructions. qPCR with specific UPL probes (Roche, http://www.roche.com) and primers designed by ProbeFinder Software was performed using a LightCycler 480 Instrument and LightCycler 480 Probes Master (Roche). The presented results are means obtained from three independent biological replicate experiments, each analysed in triplicate. Quantitative differences in transcript abundance were deemed significant if there was an absolute DEX/mock ratio ≥1.3, with *t*-test *P*-values <0.05. For details, see Supplementary Methods S1 at *JXB* online.

## Results

### Cytokinin pool dynamics following *HvCKX2* activation in *Arabidopsis* seedlings

**Fig. 1.**
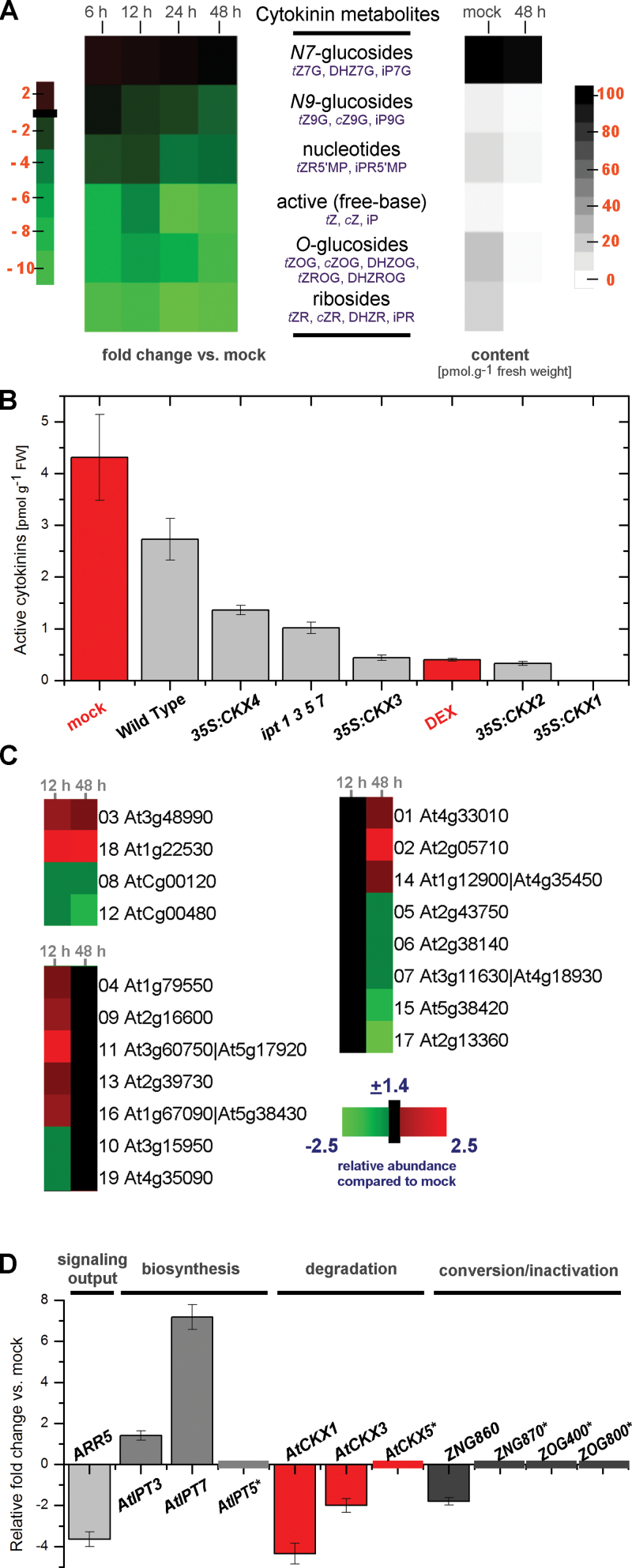
DEX-inducible *HvCKX2* expression. (A) Time course of cytokinin depletion in *CaMV35S>*GR*>HvCKX2* seedlings following *HvCKX2* activation. Seven-day-old seedlings were transferred onto MS medium supplemented with DEX or mock and samples were collected for cytokinin determination 6, 12, 24, and 48h later. (B) Comparison of CK depletion in *CaMV35S*>GR>*HvCKX2* and constitutive *35S:AtCKX* transgenics, and the quadruple *atipt1 3 5 7* mutant. Pool of active (free base) CKs; data for the *CaMV35S*>GR>*HvCKX2* line (mock, DEX) are marked in red. Data for the *35S:CKX1-35S:CKX4* and *ipt1 3 5 7* mutant used in this comparison are from 10-day-old *Arabidopsis* seedlings as reported by Nishiyama *et al.* (2011). (C) Proteins that responded to *HvCKX2* activation identified by 2-DE analysis. MALDI TOF/TOF MS was used to identify proteins in spots representing differentially regulated proteins at each of the time points. Time courses of changes in their abundance are presented as a heatmap; accession numbers are as in the TAIR database. Numbers correspond to the spot number as given in Supplementary Table S1 at *JXB* online and in the 2-DE RuBisCO-depleted proteome map (Supplementary Fig. S5). See the Supplementary data for details. (D) Relative fold change in transcripts of 10 genes involved in CK metabolism and signalling by RT–qPCR 48h after *HvCKX2* activation.

The binary pOp-HvCKX2/LhGR system of DEX-inducible *HvCKX2* expression was used to decrease endogenous CK levels of light-grown *Arabidopsis* seedlings then associated proteomic and metabolomic changes were examined. To examine CK dynamics following *HvCKX2* activation, 20 CK moieties were monitored in *CaMV35S*>GR>*HvCKX2* line 13 seedlings cultivated for 7 d on mesh on solid MS medium and subsequently incubated on mesh on the surface of MS liquid medium without (mock) or with 20 µM DEX for 6, 12, 24, and 48h (Fig. 1A; Supplementary Fig. S2 at *JXB* online). Dramatic reductions were observed in *t*Z, *c*Z, and iP levels after 6h of DEX treatment, followed by oscillations in *t*Z levels, probably reflecting the activation of homeostatic mechanisms that stabilize hormonal ratios in seedlings. Similar, but less pronounced, tendencies were also apparent for *c*Z and iP. The varied reductions in the individual CK bases were reflected in a shift in the *t*Z:*c*Z:iP ratio from 32:1.3:1 at 6h to 4.2:0.5:1 after 48h. This shift is consistent with the higher substrate preference of HvCKX2 for *t*Z than for other CK bases (Galuszka *et al.*, 2004) (for details, see Supplementary Fig. S2). Most of the CK pool consisted of biologically inactive CK metabolites, most prominently cytokinin-*N* ^7^-glucosides (CK7Gs). Unlike free CKs and most of their metabolites, the CK7G content increased by ~40% after just 6h of *HvCKX2* activation. After 48h, the CK7G content had returned to levels comparable with those of mock-treated seedlings (Supplementary Fig. S3), which still represented ~90% of the CK pool. CK7Gs are reportedly not degraded either by HvCKX2 or by endogenous AtCKXs (Galuszka *et al.*, 2007; Kowalska *et al.*, 2010). Thus, their increase probably indicates an increased rate of CK biosynthesis—a mechanism whereby plants can counteract CK degradation by HvCKX2. The CK that increased to the highest level was iP7G. Accordingly, native plant IPTs produce iP-type CKs *in vivo* (e.g. Ueda *et al.*, 2012), *t*Z is the preferred substrate for HvCKX2 (Galuzska *et al*., 2004), and CK degradation is a reaction concurrent with *N* ^*7*^-glucosylation in CK base inactivation (Brzobohatý *et al.*, 1994; Frébort *et al.*, 2011).

To obtain insights into the regulation of genes underlying homeostatic responses to CK degradation by HvCKX2, steady-state levels of transcripts of 10 genes involved in CK metabolism and signalling were examined by RT–qPCR 48h after *HvCKX2* activation (Fig. 1D; Supplementary Table S9 at *JXB* online). The reduction in free CK bases correlated with reductions in transcripts of *ARR5*, a CK primary response gene (D’Agostino *et al.*, 2000) that is frequently used to assess CK signalling. Similarly, genes involved in CK inactivation via either degradation (*AtCKX1* and *AtCKX3*) or irreversible *N*-glucosylation (*At5g05860* and *At5g05870*) were down-regulated, while expression of an *O*-glucosyltransferase gene (*At1g22400* and *At2g36800*) was unaffected. *AtIPT3* and *AtIPT7* genes were up-regulated, in accordance with the presumed increase in the CK biosynthesis rate.

To assess the extent of CK depletion in the *CaMV35S*>GR>*HvCKX2* seedlings, their measured CK contents after 48h of *HvCKX2* activation were compared with previously reported contents of seedlings constitutively ectopically overexpressing *AtCKX1*, *AtCKX2*, *AtCKX3*, and *AtCKX4* (encoding genuine *Arabidopsis* CKXs) and the CK biosynthesis-deficient *atipt1 3 5 7* mutant (Fig. 1B; Supplementary Fig. S4 at *JXB* online). CKs were depleted more strongly in the *CaMV35S*>GR>*HvCKX2* seedlings than in all the other transgenic and mutant lines except *35S:CKX1* plants.

### Proteomic and metabolomic responses to cytokinin pool alterations

To obtain insights into molecular events triggered by the CK pool alterations, associated proteomic and metabolomic responses were examined. First, proteome changes caused by HvCKX2 activity were monitored by 2-DE of RuBisCO-depleted protein extracts, followed by image and MALDI TOF/TOF MS analyses of *CaMV35S*>GR>*HvCKX2* seedlings, sampled 12h and 48h after *HvCKX2* activation (corresponding to partial recovery from the initial shock and system stabilization, respectively, according to the observed changes in *t*Z contents). A total of 748 different protein spots were detected and quantified, of which 19 (~3%) showed significant changes in relative volume compared with mock-treated samples. Seven showed a significant transient response only at 12h, eight a delayed response at 48h, and four similar, early responses at 12h and 48h of DEX treatment (Fig. 1C; Supplementary Fig. S5, Table S1 at *JXB* online).

**Fig. 2.**
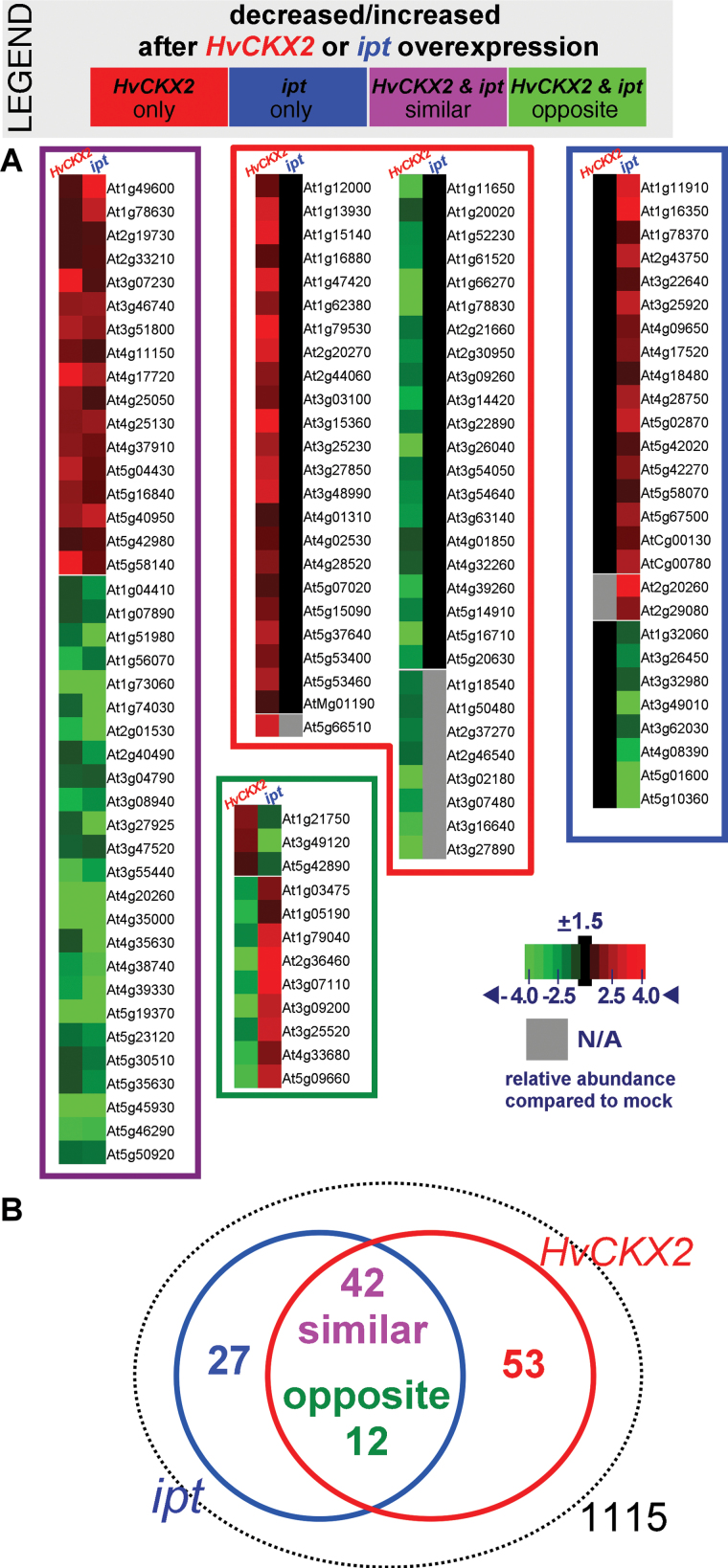
Proteins responsive to *HvCKX2* and *ipt* activation identified by gel-free LC-MS profiling. (A) Proteomes of 7-day-old *CaMV35S>*GR*>HvCKX2* and *CaMV35S>*GR*>ipt Arabidopsis* seedlings treated with DEX or mock for 48h were resolved by C18 reverse-phase liquid chromatography and analysed using an LTQ-Orbitrap XL mass spectrometer. In total, 134 proteins (~12% of all proteins meeting identification criteria) that responded to 48h of *HvCKX2* or *ipt* activation and were assembled into groups of proteins that were responsive (i) only to *HvCKX2* (*HvCKX2*; red); (ii) only to *ipt* (*ipt*; blue); or to both *HvCKX2* and *ipt* either (iii) similarly (purple) or (iv) oppositely (green). The heatmap represents fold changes in the abundance of proteins in seedlings after DEX treatment compared with the respective mock-treated control; accession numbers are as in the TAIR database. (B) Venn diagram illustrating the overlap in identified proteins. See Supplementary Table S2 at *JXB* online for detailed information on the identified proteins.

To increase proteome coverage, an LC-MS shotgun proteomic analysis of total soluble proteins was next conducted. As *HvCKX2* activation probably results in at least local increases in CK levels due to *AtIPT3* and *AtIPT7* activation, transgenic *CaMV35S*>GR>*ipt* line pOp^BK^-ipt 11 (Craft *et al.*, 2005), allowing DEX-inducible increases in CK levels, was analysed, in parallel to *CaMV35S*>GR>*HvCKX2* line 13 at 48h after DEX treatment. In total, >1700 proteins were identified in at least one of three repeated LC-MS experiments. However, only 1115 were present in all three replicates (Fig. 2; Supplementary Table S3 at *JXB* online). The comparison of normalized spectral counts of DEX- and mock-treated samples revealed 153 potentially responsive proteins. Of these, 19 were removed after manual verification of the respective peptide ion signal peak areas, and 53 significantly responded only in *CaMV35S*>GR>*HvCKX2* seedlings, about twice the number of proteins that exclusively responded to *ipt* activation. Large numbers of proteins responded to both *HvCKX2* and *ipt* activation, 42 in similar and 12 in opposite directions. HvCKX2 was detected in *CaMV35S*>GR>*HvCKX2* seedlings only upon DEX treatment (Supplementary Table S8, Fig. S9) confirming the tightness of the pOp-HvCKX2/LhGR system.

**Fig. 3.**
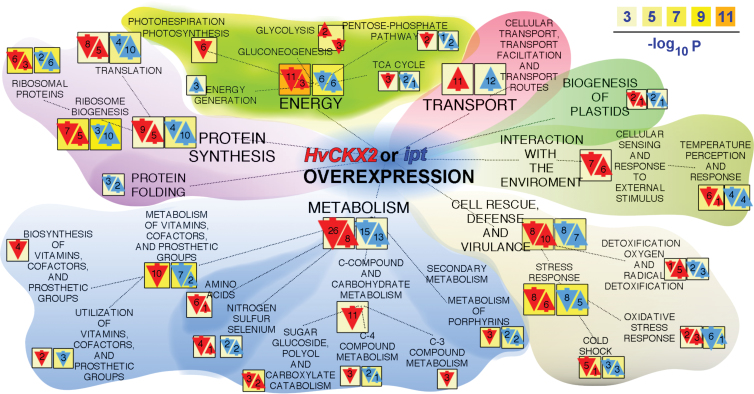
Proteins that responded to *HvCKX2* and *ipt* activation: functional classification according to the Munich Information Center for Protein Sequences (MIPS). The orientation and size of arrows indicate the direction of protein regulation (up or down) and number of proteins in a particular category, respectively. Red and blue: categories induced by *HvCKX2* and *ipt* activation; the colour scale indicates the statistical significance of each category. The BioMap tool package (http://virtualplant.bio.nyu.edu/) was used for the analysis.

The subcellular location of each identified protein was determined using the SUBA database (http://suba.plantenergy.uwa.edu.au/; Heazlewood *et al.*, 2007). The largest group of these proteins is located in chloroplasts (29.5%), followed by the plasma membrane (27%) and cytosol (11%) (Supplementary Fig. S6 at *JXB* online). Identified proteins were analysed by the BioMaps tool package (http://virtualplant.bio.nyu.edu/cgi-bin/vpweb/; Katari *et al.*, 2010). Functional classification according to MIPS (Munich Information Center for Protein Sequences) revealed that the categories ‘energy’, ‘ribosome biogenesis’, ‘metabolism of vitamins, cofactors, and prosthetic groups’, and ‘stress response’ were most significantly enriched (Fig. 3). The general categories of enriched proteins were highly similar in samples from seedlings with both increases and decreases in bulk levels of endogenous CKs, but the overlap of individual proteins differed substantially. For example, only seven out of 20 proteins in the ‘stress response’ category were enriched in both sets of samples, six of which displayed similar responses to increases and decreases in CK levels (for details, see Supplementary Table S5).

**Fig. 4.**
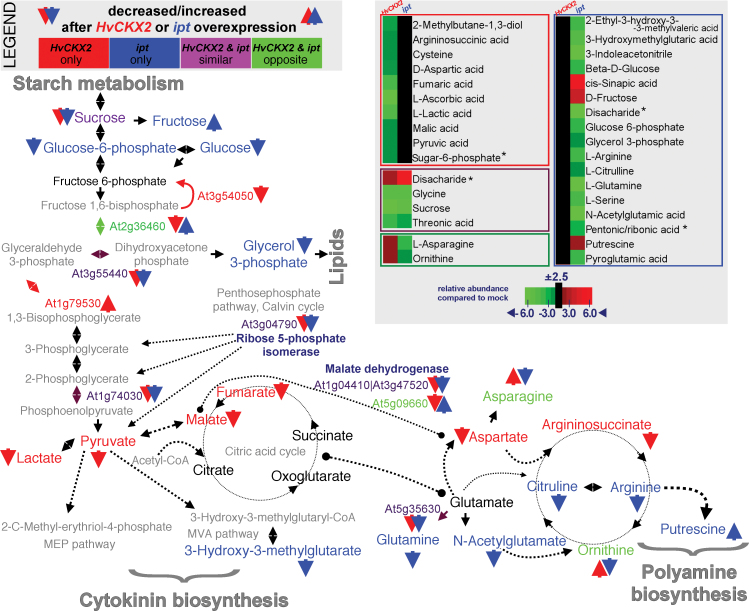
Integration of proteome and metabolome responses to *HvCKX2* and *ipt* activation. Seven-day-old *CaMV35S>*GR*>HvCKX2* (*HvCKX2*; red) and *CaMV35S>*GR*>ipt* (*ipt*, blue) *Arabidopsis* seedlings were treated with DEX or mock treated for 48h, then polar metabolites were analysed by GC-TOF-MS. In total, 33 metabolites (~41% of all annotated metabolites) significantly responded to *HvCKX2* or *ipt* activation. Integration of metabolomic and proteomic (see Figs 1 and 2) data highlighted strong responses in carbohydrate metabolism and both the citric acid and urea cycles. Arrows represent the response direction (up or down); the colour coding is explained in the key; metabolites in black were detected, but their relative abundances were not affected more than ±2.5-fold. The heatmap represents the abundance of metabolites in seedlings after DEX treatment compared with their respective mock-treated controls; (*) metabolite annotated as several similar substances.

To assess the metabolomic impact of the proteome alterations, GC-TOF-MS was used to analyse changes in metabolite levels in both *CaMV35S*>GR>*HvCKX2* and *CaMV35S*>GR>*ipt* lines following 48h of DEX treatment. The analysis resulted in a list of 79 annotated metabolites (Supplementary Table S4 at *JXB* online), including mostly amino acids, carbohydrates, and organic acids, 33 of which showed significant changes in relative abundance after DEX treatment (Fig. 4). Activation of *HvCKX2* modulated levels of 16 metabolites. Of these, glycine, sucrose, threonic acid, and an unspecified disaccharide were similarly affected after *ipt* activation, while asparagine and ornithine showed opposite responses.

## Discussion

### Proteome profiling of cytokinin down- and up-regulation

The latest version of the *Arabidopsis* genome annotation lists >27 000 protein-coding genes, and alternative splicing and post-translational modifications (PTMs) may increase the number of different proteins in biological samples to at least 100 000. Both 2-DE- and LC-MS-based proteomic experiments do not usually cover more than several thousand. Thus, in terms of coverage, proteomic techniques lag far behind genomic techniques. Nevertheless, proteomic analysis is essential for elucidating biological processes as it provides information that cannot be deduced from genomic/transcriptomic data, ranging from data on protein abundance (Baginsky *et al.*, 2010) to indications of post-translational control of protein activity (e.g. Černý *et al.*, 2010). The 2-DE and LC-MS proteomic analyses of *CaMV35S*>GR>*HvCKX2* and *CaMV35S*>GR>*ipt* seedlings covered ~6% of proteins currently deduced from the *Arabidopsis* genome. The former identified ~85% fewer proteins than the latter, which is not surprising as estimated amounts of the responsive proteins in the 2-DE Coomassie-stained spots ranged from 10ng to 600ng, far higher than the sensitivity limits of LC-MS-based analysis. Further, the overlap in responsive proteins was small; only three were detected in both data sets. Five proteins had shifted pI and/or molecular weight values, indicating the presence of PTM(s) not detected by MS. The other detected proteins did not meet criteria set for significant differences or were not found by LC-MS analysis (see 2-DE supplementary materials in Supplementary Table S1 at *JXB* online). These results confirm the benefits of 2-DE in qualitative analysis and screening for protein modifications, and imply a role for unspecified PTMs in response to CKX activity.

### Chloroplasts host the highest proportion of CK-regulated proteins

Chloroplast proteins prevailed among proteins that responded to the altered CK levels. According to a prediction algorithm developed by Kaundal *et al.* (2010), chloroplast localization is expected for 33% of all 1115 proteins identified in the LC-MS profiling, and for 45% of the responsive proteins; a significant (1.4-fold) enrichment. Similar over-representation of chloroplast proteins was found in a previous study of early CK response proteins and phosphoproteins in *Arabidopsis* (Černý *et al.*, 2011*a*), and chloroplast proteins represented a major fraction of CK-induced proteins in dark-grown *Arabidopsis* seedlings identified by Lochmanová *et al*. (2008). Accordingly, chloroplasts have been linked to CK action on numerous levels. For example, a dynamic CK pool is reportedly present in chloroplasts (Benková *et al.*, 1999) that is partly autonomous from the bulk cell CK pool (Polanská *et al.*, 2007). Further, compartmentation into chloroplasts of some CK biosynthesis and metabolism pathways has been revealed (Brzobohatý *et al.*, 1993; Kristoffersen *et al*., 2000; Takei *et al.*, 2004; Kiran *et al.*, 2006).

### Previously reported and novel links in CK signalling

The LC-MS and 2-DE analyses identified 155 proteins that responded to the altered levels of endogenous CKs in the seedlings, 73 of which have been previously implicated in plant hormone, mostly CK (58), responses in large-scale transcriptomic and proteomic analyses (Rashotte *et al.*, 2003; Brenner *et al.*, 2005; Kiba *et al.*, 2005; Nemhauser *et al.*, 2006; Lochmanová *et al.*, 2008; Chen *et al.*, 2010; Černý *et al.*, 2011*a*; Brenner and Schmülling, 2012; Nishiyama *et al.*, 2012). Direct comparison of data sets reported by different groups is not straightforward due to differences in experimental set-ups. Nevertheless, 34 proteins in the sets reported here showed similar responses to those observed in previous studies. In the set of responsive proteins yielded by LC-MS profiling, 12 displayed opposite responses to *HvCKX2* and *ipt* activation, and thus may be indicative of bulk endogenous CK levels (Table 1). Of those 12 proteins, six are novel CK-response proteins: three ribosomal proteins, a key enzyme in haem biosynthesis (coproporphyrinogen oxidase, At1g03475), a peroxidase (At3g49120) reportedly involved in cell elongation (Passardi *et al.*, 2005), and a sterol carrier protein (At5g42890) with a loss-of-function mutation phenotype correlating with known CK effects on root elongation, together with small cotyledon rosettes and short hypocotyls (Zheng *et al.*, 2008). The other six proteins have already been linked to CK responses (Brenner *et al.*, 2005; Černý *et al.*, 2011*a*; Brenner and Schmülling, 2012) and include, for example, an enzyme involved in lysine biosynthesis (At4g33680) and a peroxisomal malate dehydrogenase (At5g09660), mutation of which results, respectively, in aberrant growth and cell death (Song *et al.*, 2004), and reduced growth rates in the dark (Pracharoenwattana *et al.*, 2007). A recently assembled data set of genes with known loss-of-function mutant phenotypes in *Arabidopsis* (Lloyd and Meinke, 2012) provides information on biological functions of a further 41 proteins found here to respond to alterations in CK levels. Most of them (70%) are also essential for growth and development, including 15 for which loss of function is lethal or leads to embryo defects. Mutations in another 10 and eight of the proteins have been linked to defects in photosynthesis or biosynthesis of photosynthetic pigments and enhanced sensitivity to abiotic stresses, respectively (for details, see Supplementary Table S6 at *JXB* online).

**Table 1.** Proteins and metabolites indicative of endogenous cytokinin levels

ID (AGI/KEGG)	Recommended name	Correlation with cytokinin levels
At1g03475	Coproporphyrinogen-III oxidase, chloroplastic	Positive
At1g05190	50S ribosomal protein L6, chloroplastic	Positive
At1g79040	Photosystem II 10kDa polypeptide, chloroplastic	Positive
At2g36460	Fructose-bisphosphate aldolase	Positive
At3g07110	60S ribosomal protein L13a-1	Positive
At3g09200	60S acidic ribosomal protein P0-2	Positive
At3g25520	60S ribosomal protein L5-1	Positive
At4g33680	LL-diaminopimelate aminotransferase, chloroplastic	Positive
At5g09660	Malate dehydrogenase, glyoxysomal	Positive
At1g21750	Protein disulphide isomerase-like 1-1	Negative
At3g49120	Peroxidase 34	Negative
At5g42890	Sterol carrier protein 2	Negative
C00077	Ornithine	Negative
C00152	l-Asparagine	Negative

### ABA and CK cross-talk

Seventeen of the CK-responsive proteins identified here are abscisic acid (ABA) responsive, according to an analysis of overlaps in transcriptional responses to individual plant hormones by Nemhauser *et al.* (2006), who found 126 genes that are influenced by both CK and ABA (representing 46.8% and 4.3% of all identified CK- and ABA-modulated genes, respectively). A further 55 are ABA responsive according to data obtained from analyses of ABA-induced transcriptomic changes by Xin *et al.* (2005) and Matsui *et al.* (2008). These findings are consistent with the apparent involvement of ABA and CK cross-talk in several physiological processes, including stress responses (Tran *et al.*, 2010; Ha *et al.*, 2012) and seed germination (Wang *et al.*, 2011). Furthermore, Nishiyama *et al.* (2011) found that CK deficiency decreases ABA content, but induces ABA hypersensitivity, which may be reflected in the up- or down-regulation of 36 proteins following *HvCKX2* activation whose respective transcripts reportedly showed similar responses after plants were treated with exogenous ABA (Supplementary Table S7 at *JXB* online). In addition, seven proteins that showed no significant response to *HvCKX2* overexpression are inversely up- or down-regulated in seedlings with increased levels of endogenous CK compared with ABA-treated plants (Supplementary Table S7). Proteins that were identified as CK responsive that are apparently involved in ABA–CK cross-talk include several well-known ABA response proteins, for example magnesium-chelatase subunit chlI (At4g18480; increased in response to *ipt* activation) and RNA-binding protein At2g21660 (decreased in response to *HvCKX2* activation; mutant sensitive to ABA). They also included two proteins likely to be involved in ABA signalling (Yan *et al.*, 2009): At3g26450 (decreased in response to *ipt* activation), which contains a ligand-binding domain similar to PYR/PYL/RCAR, and mitochondrial porin (At5g67500; increased in response to *ipt* activation).

### Ethylene and CK cross-talk

Manipulation of endogenous CK levels resulted in increased abundance of two RNA-binding proteins (At4g17720 and At5g04430) previously identified in a phosphoproteomics study of ethylene responses (Li *et al.*, 2009). HvCKX2 activity up-regulated a key enzyme in ethylene biosynthesis, ACC oxidase 2 (At1g62380), and affected a number of proteins previously found to be affected in ethylene-treated seedlings (Chen *et al.*, 2011). In total, 18 proteins detected by Chen *et al.*, were identified, 14 of which responded to *HvCKX2* activation, five in the same direction as when seedlings were treated with ethylene. *Ipt* activation had milder effects, influencing three of 11 common proteins in the same manner as ethylene treatment (Supplementary Table S7 at *JXB* online). These findings indicate previously unrecognized links between ethylene and CK action or an alternative ethylene-independent regulation of the proteins originally recognized as ethylene responsive. The latter possibility is consistent with the finding that five of these proteins are also apparently ABA responsive.

### Jasmonate signalling

Jasmonates are plant hormones that are mainly involved in plant responses to biotic or abiotic stresses. Jasmonate treatments reportedly have profound effects on plant growth, including reductions in the size of leaves and root systems (e.g. Zhang and Turner, 2008). Brenner *et al.* (2012) found that 20 genes involved in CK metabolism and signalling that were most strongly induced by CK treatment were rapidly down-regulated in response to methyl jasmonate (MeJA). However, this CK deficiency-like response to MeJA is not reflected in MeJA-responsive proteins in the present data set. HvCKX2 activity increased the amount of an uncharacterized protein, At1g13930, and thylakoid lumenal protein (At4g02530), while decreasing levels of β-glucosidase (At3g09260), ATP sulphurylase (At3g22890), tryptophan synthase (At4g01850), and phosphoserine aminotransferase (At4g35630). These are opposite effects compared with those of MeJA treatment (Nagano *et al.*, 2005; Nemhauser *et al.*, 2006; Jung *et al*., 2007*a*, *b*), indicating yet more complex interactions between the two plant hormones.

### Redox-regulatory network responses to CK

Redox-regulatory components were over-represented in the present set of CK-responsive proteins. They included thioredoxin 3 (increased following activation of both *ipt* and *HvCKX2*), thioredoxin M and glutaredoxin (up-regulated in *CaMV35S*>GR>*HvCKX2* seedlings), a ferredoxin superfamily protein (down-regulated in *CaMV35S*>GR>*HvCKX2* seedlings), and 15 other proteins that are targets of thioredoxin regulation. Recently, a role for thioredoxin M in Mg chelatase regulation was indicated (Luo *et al.*, 2012). Thus, thioredoxin signalling might provide a connection between CK action and ABA signalling. Further, CK-dependent regulation of the thioredoxin regulatory network could integrate CK and light signalling in chloroplasts. This is consistent with the known CK involvement in partial chloroplast biogenesis in dark-grown *Arabidopsis* (Chory *et al.*, 1994; Lochmanová *et al.*, 2008) and over-representation of chloroplast proteins among cytokinin-responsive proteins revealed in this study and previous studies by Černý *et al*. (2011*a*) and Lochmanová *et al*. (2008).

### Ribosomal proteins: an information hub for cytokinin signalling?

Extensive regulation of transcripts encoding ribosomal proteins in response to CK was recently reported by Brenner and Schmülling (2012). A total of 76 ribosomal proteins were detected, 21% of which responded to altered CK levels (Supplementary Fig. S7 at *JXB* online). Functional classification ‘Ribosome biogenesis’ was also strongly represented, by 12 and 13 proteins that responded to *HvCKX2* and *ipt* activation, respectively (Fig. 3). Four and four proteins in the two groups displayed identical and opposite responses, respectively. Phenotypically, CKs are known to alter leaf development and morphology (Werner *et al.*, 2003; Hay and Tsiantis, 2010; Shani *et al.*, 2010), and the differentially regulated ribosomal proteins are probably involved in the underlying molecular mechanism. In support of this hypothesis, Horiguchi *et al.* (2011) showed that ribosomal proteins significantly contribute to *Arabidopsis* leaf development. In addition, an L5e mutant (At3g25520; decreased and increased following *HvCKX2* and *ipt* activation, respectively) has slightly pointed, serrated leaves, while an L4e mutant (At5g02870), which increased following *ipt* activation and is implicated in auxin responses (Rosado *et al.*, 2010), has narrow, pointed first true leaves, short roots, retarded growth, and late flowering.

### Urea cycle metabolites reflect likely cytokinin-dependent polyamine biosynthesis

The urea cycle is one of the few points unequivocally distinguishing *ipt* and *HvCKX2* action. Decreases in arginine, citrulline and ornithine were observed following *ipt* activation, probably resulting from enhanced polyamine biosynthesis, which is reportedly triggered by increased CK levels (e.g. Walker *et al.*, 1988) and is indicated in the present data set by an increase in the polyamine putrescine. The depletion in urea cycle metabolites provides an explanation for reductions observed in glutamine and other glutamate derivatives, which is also consistent with down-regulation of glutamine synthetase. Thus, glutamate is probably predominantly converted to citrulline directly or indirectly through its *N*-acetyl group to ornithine. *HvCKX2* activation led to increased ornithine levels, possibly at the expense of argininosuccinate and aspartate pools. The ornithine accumulation indicates a decrease in cytokinin-dependent conversion of arginine to polyamines and that ornithine may be a possible marker of endogenous CK levels in the cell.

### Pyruvate: a pivotal mediator of ipt and HvCKX2 action

Integration of the proteomic and metabolomic data highlighted complex responses of carbohydrate metabolism to the manipulations of endogenous CK levels. Demands on the sucrose pool clearly rose following activation of both *ipt* and *HvCKX2*, probably at the expense of starch biosynthesis via a mechanism that maintains the supply of pyruvate. IPT is reportedly specialized for plastid localization and preferential use of 1-hydroxy-2-methyl-2-(*E*)-butenyl 4-diphosphate (HMBDP) as a prenyl donor *in vivo* (Ueda *et al.*, 2012). In plastids, HMBDP is generated via the methylerythritol phosphate (MEP) pathway starting from pyruvate. It was previously found that pyruvate homeostatic mechanisms are sufficient to cope with increases in *t*-ZMP amounting to >10 nmol g^–1^ fresh weight in DEX-treated *CaMV35S*>GR>*ipt* seedlings (Hradilová *et al.*, 2007). Increased pyruvate consumption is apparently compensated by increased glycolysis, as indicated by the observed depletion of sucrose, glucose, and glucose-6-phosphate. Interestingly, a >4-fold decrease was found in 3-hydroxy-3-methylglutarate, which is linked to a mevalonate pathway that generates prenyl donors in the cytosol, indicating that IPT may have unrecorded activity in the cytosol or that cytosolic isopentenyl pyrophosphate and plastid HMBDP levels may be connected by an unknown mechanism. Alternatively, CK might stimulate biosynthesis of an uncharacterized product originating from the terpenoid biosynthetic pathway. Pyruvate depletion following *HvCKX2* activation could result from the increase in ornithine synthesis deduced from the metabolomic data set. An apparent local increase in CK biosynthesis in activated *CaMV35S*>GR>*HvCKX2* seedlings (indicated by up-regulation of *AtIPT3* and *AtIPT7*) would also increase demands on the pyruvate pool, but this would be unlikely to result in a detectable decrease in bulk pyruvate as pyruvate levels were not affected in activated *CaMV35S*>GR>*ipt* seedlings.

### Cytokinin homeostatic mechanisms may account for significant similarities in phenotypes resulting from down- and up-regulation of bulk cytokinin levels

**Fig. 5.**
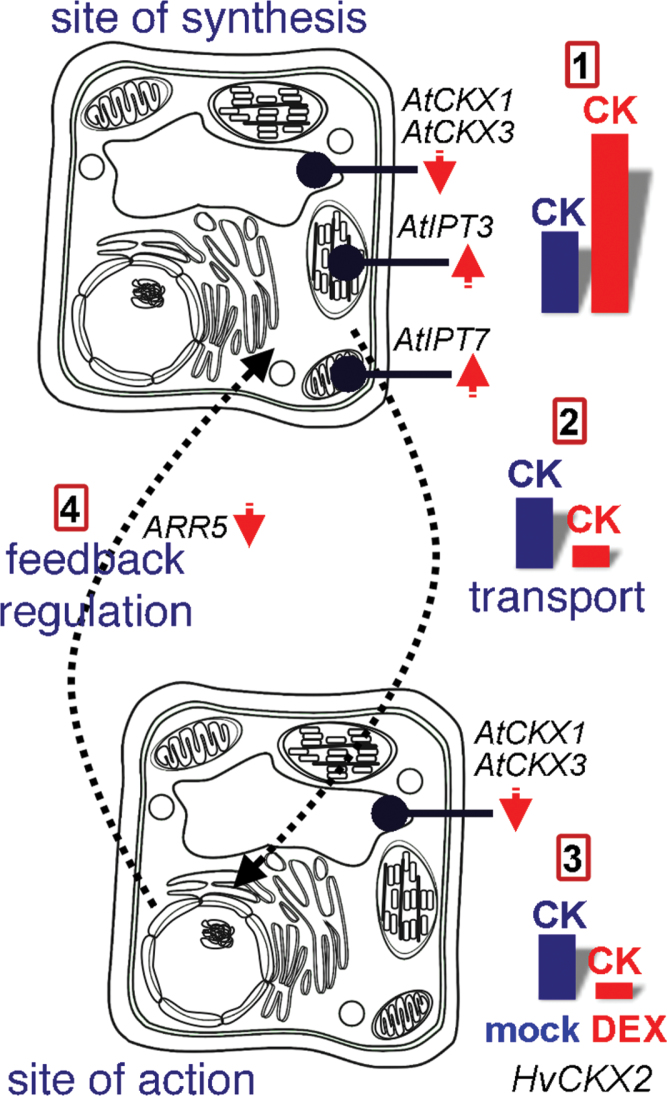
Hypothetical model of local CK increases in response to *HvCKX2* activation. From a synthesis site (1), CKs are transported (2) to sites of action (3), and the synthesis rate is regulated by homeostasis-maintaining mechanisms (4). The closest HvCKX2 homologue in rice is apoplastic OsCKX7, and recent experiments have shown that HvCKX2 is predominantly localized in the leaf vasculature (P. Galuszka, unpublished results). Thus, HvCKX2 is a potent molecular tool for disrupting CK transport and (hence) decreasing cellular contents of active cytokinins, as evidenced by the down-regulation of a CK primary response gene *ARR5*, and *AtCKX1* and *AtCKX3* genes involved in CK down-regulation under standard conditions. In turn, CK biosynthesis is increased at synthesis sites via activation of *AtIPT* genes. Here, *HvCKX2* activation resulted in up-regulation of *AtIPT3* and *AtIPT7*, two of the three major *AtIPT* genes (Takei *et al.*, 2004), of which *AtIPT3* is the most prominent in shoots and the second most abundant in roots.

Up- and down-regulation of CK levels has been used to modulate a number of biological processes and engineer agriculturally important traits. However, comparison of effects of up- and down-regulation of CK levels on a particular biological process has occasionally resulted in seemingly conflicting conclusions. For example, up-regulation of CK production is reportedly an important factor in adaptation to salt stress in tomato and maize (Vyroubalová *et al.*, 2009; Ghanem *et al.*, 2011). However, cytokinin-deficient *Arabidopsis* plants are also reportedly strongly salt stress tolerant (Nishiyama *et al.*, 2011). In the seedlings examined here, *ipt* and *HvCKX2* activation yielded overlaps, of 31% and 12% of responsive proteins and metabolites, respectively, which cannot be explained solely as a general stress response. Fourteen of the overlapping proteins have been previously identified as cytokinin responsive, and the STRING (http://string-db.org; Szklarczyk *et al.*, 2011) protein–protein interaction network analysis indicates that proteins in the overlapping set are associated with diverse processes, including chlorophyll biosynthesis, carbohydrate metabolism, thioredoxin regulation, protein transport, proteasome-mediated degradation, and ribosome biogenesis (Supplementary Fig. S8 at *JXB* online). A decrease in the bulk CK pool via HvCKX2 activity results in attenuation of cytokinin signalling (manifested here in down-regulation of *ARR5* transcripts) which, in turn, induces *de novo* CK biosynthesis via a CK homeostatic mechanism, manifested by up-regulation of *AtIPT3* and *AtIPT7* transcripts (Fig. 5). This is likely to generate a local increase in CK contents at CK biosynthesis sites, as manifested by the increased bulk content of CK7Gs (non-degradable products of irreversible CK inactivation). Interestingly, increased CK contents have been found in *Arabidopsis* CK receptor mutants, indicating a homeostatic control of steady-state CK levels through signalling (Riefler *et al.*, 2006).

Local increases in CK contents might at least partially explain the similarities in responses to *HvCKX2* and *ipt* activation observed for 42 of the identified proteins. Similarly, both *ipt* activation and CK treatment can result in increases in *AtCKX* transcripts (Hoth *et al.*, 2003; Rashotte *et al.*, 2003; Brenner *et al.*, 2005), which might lead to a local drop in CK content. Thus, local reductions in CK activity may follow *ipt* activation, which might, likewise, contribute to the similarity in observed responses to *HvCKX2* and *ipt* activation. In this light, the apparent conflicts in the results of salinity stress experiments could be interpreted as follows. Salinity stress may be accompanied by an increase in CK biosynthesis. Rates of CK biosynthesis increase and CK signalling outputs are adjusted in plants with increased CKX and IPT activities, via mechanisms that are used to cope with environmental stresses. Such mechanisms would also explain the beneficial effects of exogenous CK application to wheat plants under high salinity conditions (Gadallah, 1999).

### Conclusion

The comparative proteome- and metabolome-wide analysis of molecular events triggered by induced reductions and increases in bulk CK levels resulted in identification of numerous novel CK-responsive proteins and metabolites, including several that are potential reporters of up- and down-regulation of CK activity *in planta*. The data obtained fundamentally deepen our understanding of the roles CK in thioredoxin signalling and ribosome biogenesis, as well as cross-talk between CK and stress-related hormones. Further, the comparative analysis provided novel indications that CK homeostatic mechanisms may explain significant similarities in phenotypes resulting from reductions and increases in bulk CK levels, *inter alia* the highly similar responses to salinity stress of plants with both increased and decreased CK levels. The findings also highlight previously unrecognized challenges facing attempts to construct transgenic plants with improved temporal and spatial targeting of CK metabolism genes in order to enhance key agricultural traits including yield improvements and stress tolerance.

## Supplementary data

Supplementary data are available at *JXB* online.

Figure S1. Schematic diagram of pOpOn2.1::gHvCKX2.

Figure S2. Time course of changes in active cytokinin contents in *CaMV35S*>GR>*HvCKX2* seedlings following *HvCKX2* activation.

Figure S3. Time course of change in CK conjugate contents in *CaMV35S*>GR>*HvCKX2* seedlings following *HvCKX2* activation.

Figure S4. Comparison of cytokinin depletion in *CaMV35S*>GR>*HvCKX2* and constitutive *35S:AtCKX* transgenics, and the quadruple *atipt1 3 5 7* mutant.

Figure S5. Effects of *HvCKX2* activation on the proteome of *Arabidopsis* seedlings.

Figure S6. Subcellular distributions of the differentially regulated proteins according to predictions and SUBA experimental data.

Figure S7. Ribosomal proteins that responded to *HvCKX2* and *ipt* activation.

Figure S8. Protein–protein interaction network constructed using STRING.

Figure S9. Expression of *ipt* is evident, but not detectable at the protein level.

Table S1. 2-DE-based analysis.

Table S2. LC-MS proteome analysis: differentially regulated proteins.

Table S3. LC-MS proteome analysis: list of all identified proteins.

Table S4. GC-MS metabolome analysis.

Table S5. BioMaps results.

Table S6. Known loss-of-function mutant phenotypes for the differentially regulated proteins found by 2-DE and LC-MS profiling.

Table S7. Overlaps of cytokinin-responsive proteins and transcripts detected here and in previous proteomic and transcriptomic analyses of hormone action in *Arabidopsis*.

Table S8. Detectability of HvCKX2 and ipt in the LC-MS data.

Table S9. Steady-state levels of transcripts of 10 genes involved in CK metabolism and signalling by RT–qPCR 48h after *HvCKX2* activation.

Methods S1. Overview of the experiments and supplementary Materials and methods

Supplementary Data
