# Supplementary material for: Proteome and metabolome profiling of cytokinin action in Arabidopsis identifying both distinct and similar responses to cytokinin down- and up-regulation
Source: J Exp Bot. 2013 Sep 24;64(14):4193–206. doi: 10.1093/jxb/ert227 (PMC3808309; doi:10.1093/jxb/ert227)
Supplement: Supplementary Data [file supp_64_14_4193__index.html]

Proteome and metabolome profiling of cytokinin action in Arabidopsis identifying both distinct and similar responses to cytokinin down- and up-regulation — Proteome and metabolome profiling of cytokinin action in Arabidopsis identifying both distinct and similar responses to cytokinin down- and up-regulation — Supplementary Data 

# Proteome and metabolome profiling of cytokinin action in *Arabidopsis* identifying both distinct and similar responses to cytokinin down- and up-regulation

## 

Data files

**Files in this Data Supplement:**

- Supplementary Data - Supplementary Data
- Supplementary Data - Supplementary Data
- Supplementary Data - Supplementary Data
