# Supplementary material for: Proteome and metabolome profiling of cytokinin action in Arabidopsis identifying both distinct and similar responses to cytokinin down- and up-regulation
Source: J Exp Bot. 2013 Sep 24;64(14):4193–206. doi: 10.1093/jxb/ert227 (PMC3808309; doi:10.1093/jxb/ert227)
Supplement: Supplementary Data [file supp_ert227_jexbot098541_file001.pdf]

Proteome and metabolome profiling of cytokinin action in Arabidopsis identifying both distinct and similar responses to cytokinin down- and up-regulation

Martin Černý, Alena Kuklová, Wolfgang Hoehenwarter, Lena Fragner, Ondřej Novák, Gabriela Rotková, Petr L. Jedelský, Kateřina Žáková, Mária Šmehilová, Miroslav Strnad, Wolfram Weckwerth, Břetislav Brzobohatý

Figures S1-S9

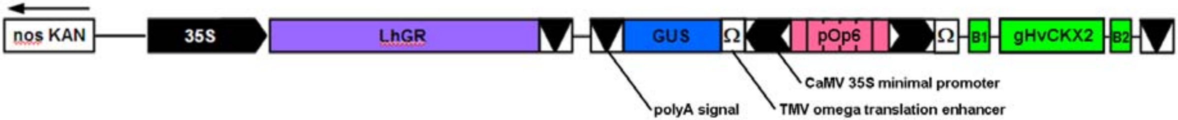

Fig. S1 Schematic diagram of pOpOn2.1::gHvCKX2. The construct represent binary T-DNA vector where right T-DNA border is located to the right of the diagram. The gene of interest is colored green; nos-KAN - kanamycin resistance gene under control of nopaline synthase promoter; 35S - CaMV 35S promoter, LhGR (Wielopolska *et al*., 2005) - glucocorticoid (DEX) dependent transcription factor; pOp6 - promoter that consists of six copies of an optimized *lac* operator sequence (Šámalová *et al.*, 2005); other regulatory sequences are described in abovementioned publications.

Šámalová M, Brzobohatý B, Moore I. 2005. pOp6/LhGR: a stringently regulated and highly responsive dexamethasone-inducible gene expression system for tobacco. *The Plant Journal* **41**, 919–935.

Wielopolska A, Townley H, Moore I, Waterhouse P, Helliwell C. 2005. A high-throughput inducible RNAi vector for plants. *Plant Biotechnology Journal* **3**, 583–590.

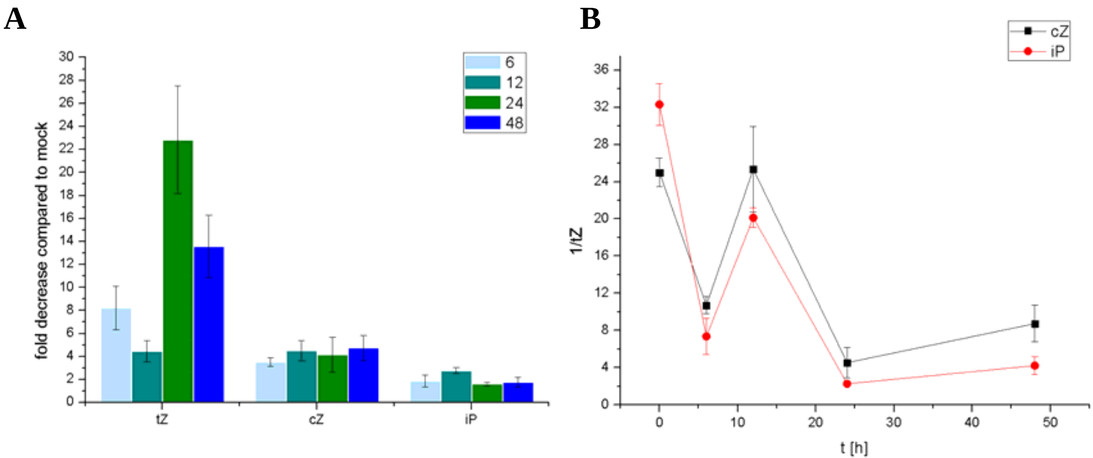

Fig. S2 Time course of changes in active CK contents in *CaMV35S*>GR>*HvCKX2* seedlings following *HvCKX2* activation.

7-day-old *CaMV35S*>GR>*HvCKX2* seedlings were treated with DEX and their CK contents were determined after 6, 12, 24 and 48 h of DEX treatment.

(A) Decrease in *trans*-zeatin (tZ), *cis*-zeatin (cZ) and isopentenyl adenine (iP);

(B) Relative content of tZ compared to cZ (black) and iP (red).

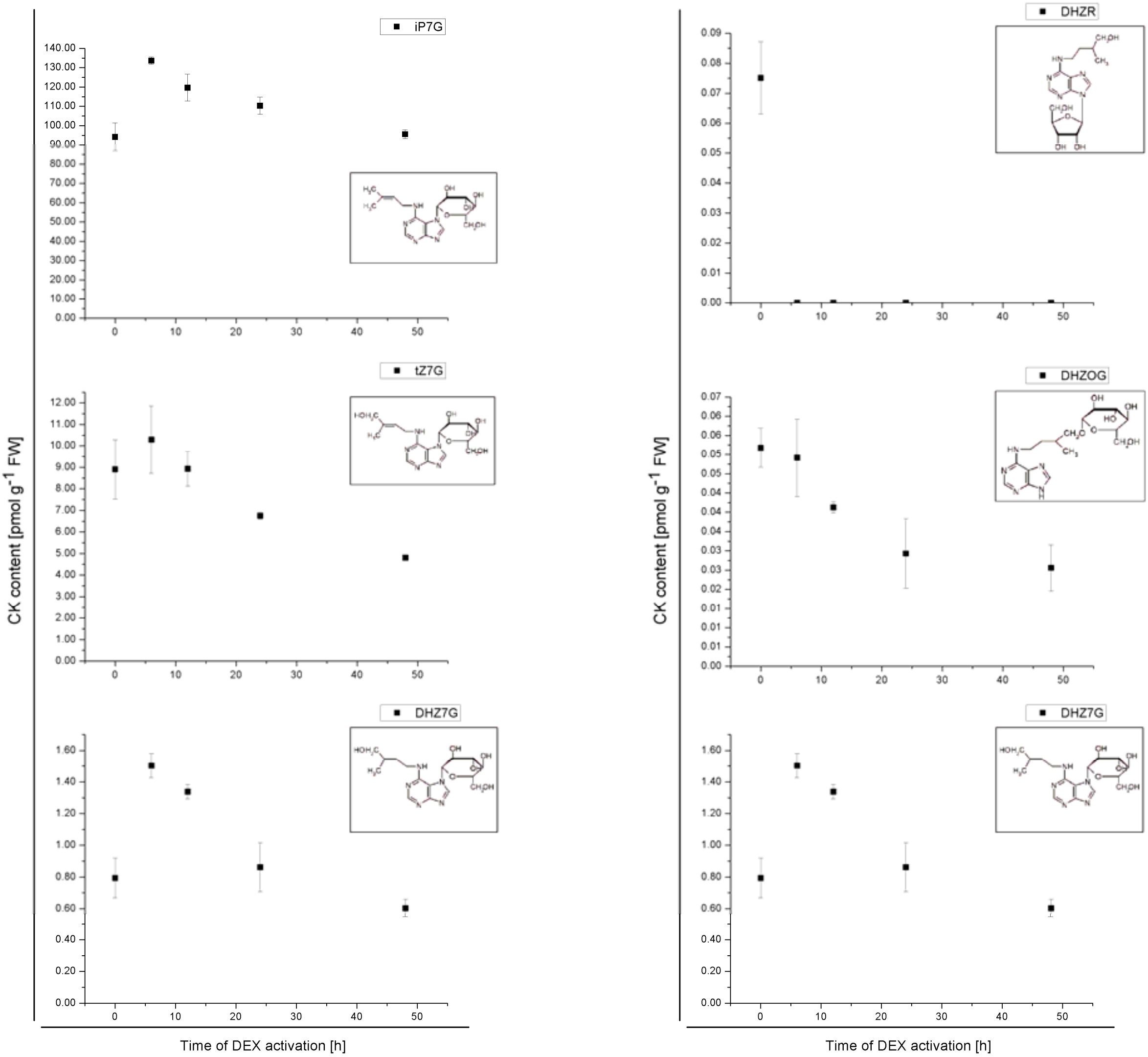

Fig. S3 Time course of changes in CK conjugate contents in *CaMV35S*>GR>*HvCKX2* seedlings following *HvCKX2* activation.

7-day-old *CaMV35S*>GR>*HvCKX2* seedlings were treated with DEX and their CK conjugate contents were determined after 6, 12, 24 and 48 h of DEX treatment.

iP7G – isopentenyladenine-*N7*-glucoside, tZ7G – *trans*-zeatin-*N7*-glucoside, DHZ7G – dihydrozeatin-*N7*-glucoside, DHZR – dihydrozeatin-*N9*-riboside, DHZOG – dihydrozeatin-*O*-glucoside.

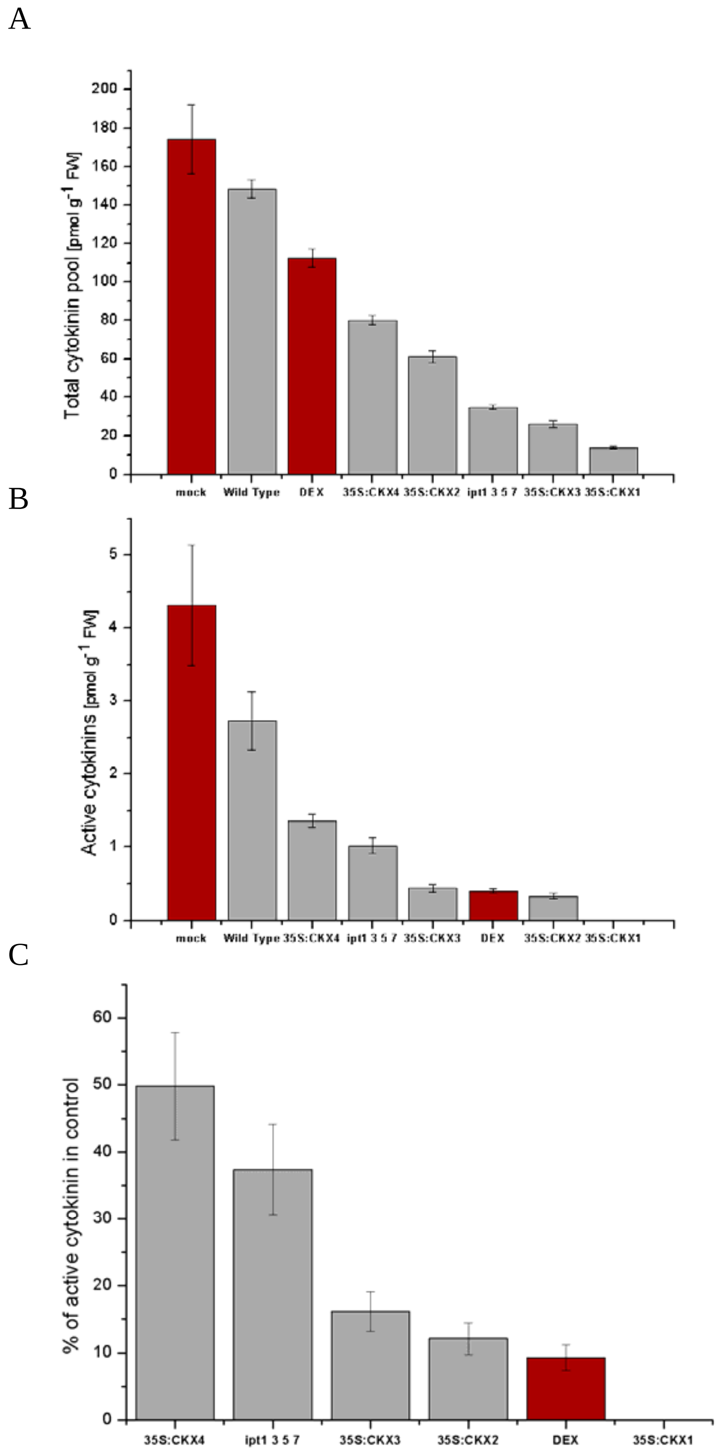

Fig. S4 Comparison of CK depletion in *CaMV35S*>GR>*HvCKX2* and constitutive *35S:AtCKX* transgenics, and the quadruple *atipt1 3 5 7* mutant.
(A) Total CK pool; **(B)** Pool of active (free base) CKs; **(C)** Active CK pool in *ipt 1 3 5 7* mutant and *CKX* transgenics relative to wild-type (or mock). Data for the *CaMV35S*>GR>*HvCKX2* line (mock, DEX) marked in red. Data for the *35S:CKX1-35S:CKX4* and *ipt1 3 5 7* mutant used in this comparison are from 10-day-old Arabidopsis seedlings as reported by Nishiyama *et al.* (2011).

Nishiyama R, Watanabe Y, Fujita Y, Le DT, Kojima M, Werner T, Vankova R, Yamaguchi-Shinozaki K, Shinozaki K, Kakimoto T, Sakakibara H, Schmülling T, Tran LP. 2011. Analysis of cytokinin mutants and regulation of cytokinin metabolic genes reveals important regulatory roles of cytokinins in drought, salt and abscisic acid responses, and abscisic acid biosynthesis. *The Plant Cell* **23**, 2169-2183.

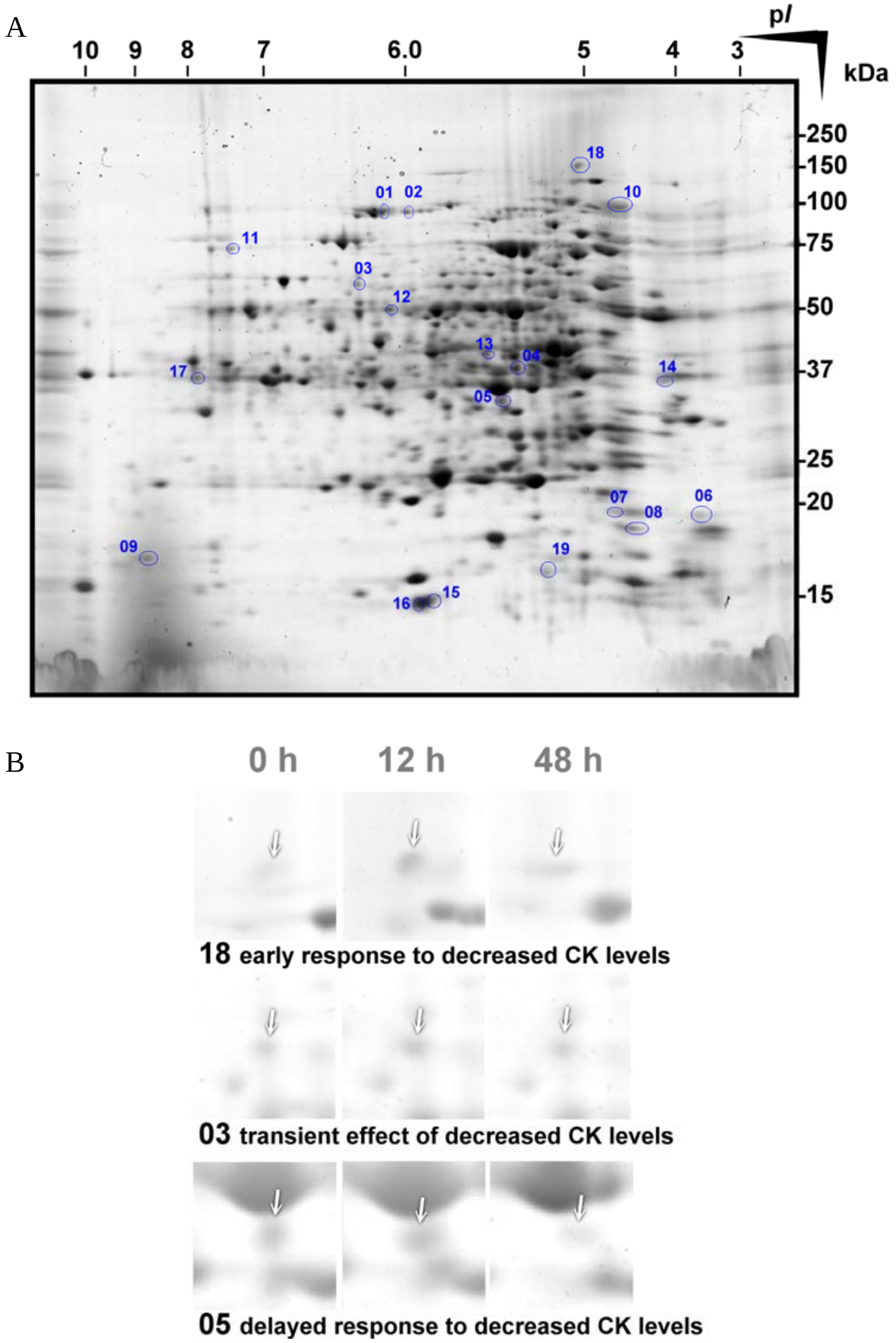

Fig. S5 Effects of *HvCKX2* activation on the proteome of Arabidopsis seedlings. Seedlings were treated as in (Fig. 1A – hormonal analysis) and samples collected after 0, 12 and 48 h of DEX/mock treatment. (A) average two-dimensional gel electrophoresis RuBisCO-depleted proteome map of the seedlings. Spots representing differentially regulated proteins are indicated. Proteins (150 μg) were separated by IPG (7 cm strips, pH 3-10NL) followed by 8-20% SDS–PAGE then visualized by Bio-Safe Coomassie G250 staining. Isoelectric points (pI) and migrating positions of molecular mass (kDa) markers are indicated. PAGE background was compensated by Decodon Delta 2D to optimize visibility of all protein spots. For details, see *Experimental procedures*. **(B)** examples of regions of 2D gels showing protein responses to *HvCKX2* activation. See Table S1 for details.

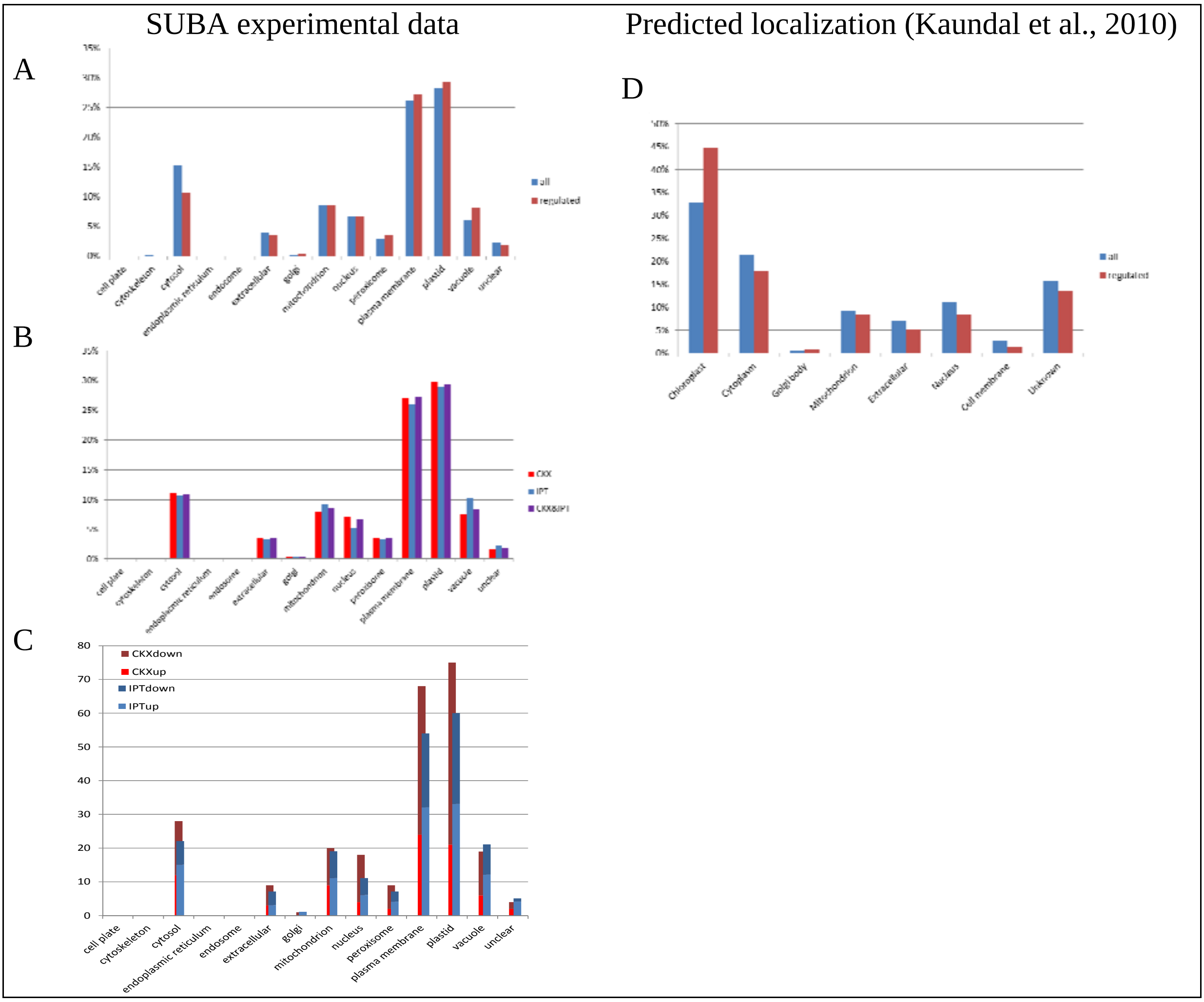

Fig. S6 Subcellular distributions of the differentially regulated proteins according to predictions and SUBA experimental data

(A) Subcellular distributions of identified proteins. The subcellular location of each identified protein was determined according to the SUBA database [http://suba.plantenergy.uwa.edu.au/; (Heazlewood et al., 2007)]. The SUBA experimental dataset from large-scale proteomic and GFP localization includes information on 126 (94 %) of the differentially regulated proteins (‘regulated’, red) and 961 (86 %) of all proteins identified in this work (‘all’, blue). The largest group of the differentially regulated proteins is localized to chloroplasts (29.5 %), followed by the plasma membrane (27 %) and cytosol (11 %). The results indicate significant depletion of cytosolic proteins (-1.43), enrichment of vacuolar proteins (+1.38), and weak enrichment of plastid and plasma membrane proteins (+1.04) in the regulated proteins. However, clear evidence based on GFP localization was obtained for only ~ 27 % of identified proteins and many proteomic-based annotations are conflicting, resulting in 1.9 localizations on average for each protein.

(B) Subcellular distributions of the differentially regulated proteins and **(C)** total number of their respective localization annotations. The compartmentalization of proteins responding to only *HvCKX2* (‘CKX’, red) or only *ipt* (‘IPT’, blue) activation, and those responding to both *HvCKX2* and *ipt* (‘CKX&IPT, violet) activation is similar.

(D) The prediction algorithm by Kaundal et al. (2010) estimates chloroplast localization for 45 % of our regulated proteins, but only 33 % of the whole set of identified proteins, representing significant (1.4-fold) enrichment, accompanied with reductions in all other major categories.

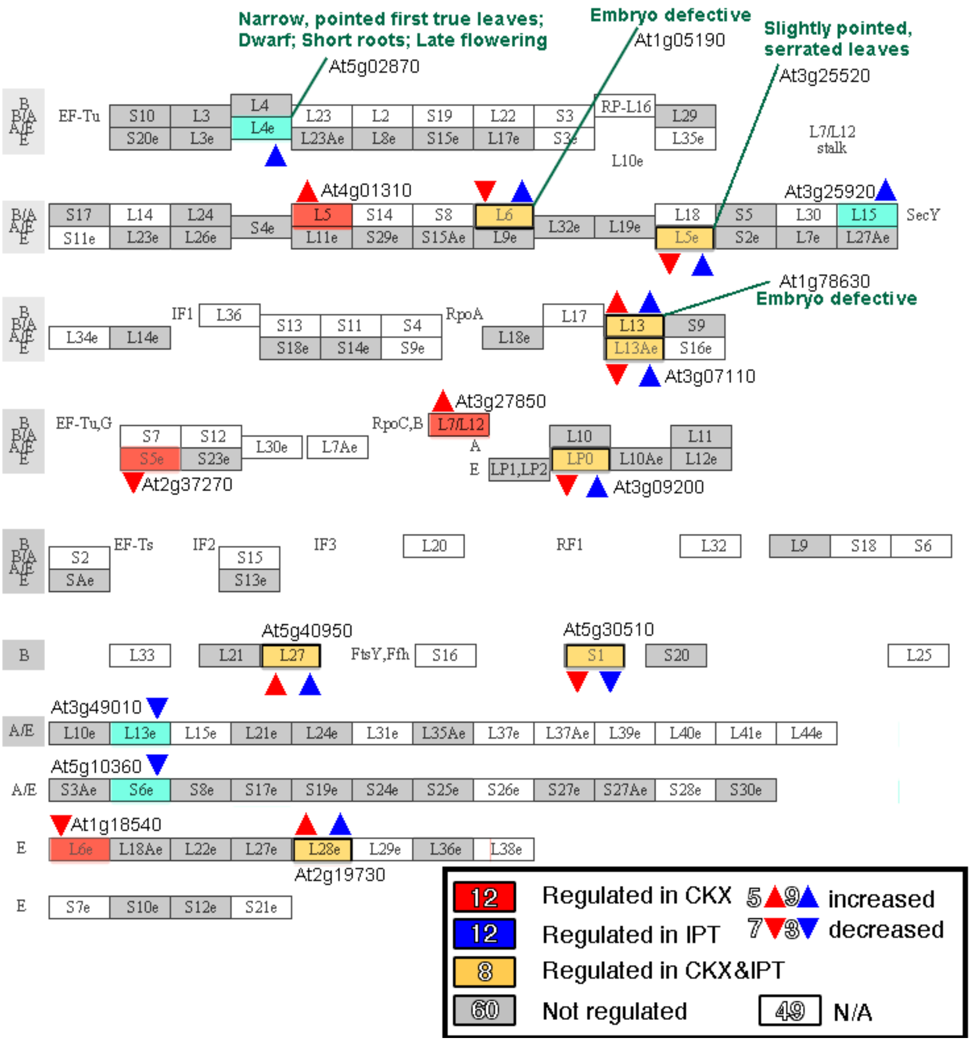

Fig. S7 Ribosomal proteins that responded to *HvCKX2* and *ipt* activation. Functional classification showed that the category 'ribosome biogenesis' was strongly represented in the responsive proteins: 12 and 13 proteins responded to *HvCKX2* and *ipt* activation, respectively (Figure 3). Eight of these proteins showed similar and four opposite responses. Further analysis of the proteomic results revealed that 21 % of 76 detectable ribosomal proteins in the Arabidopsis ribosome are affected by modulated cytokinin levels. Sixty detected proteins were not significantly affected by either *HvCKX2* or *ipt* activation (gray), and levels of 49 were below the detection limits. Notes in green indicate a loss-of-function mutant phenotype in Arabidopsis as annotated in the database recently compiled by Lloyd and Meinke (2012).

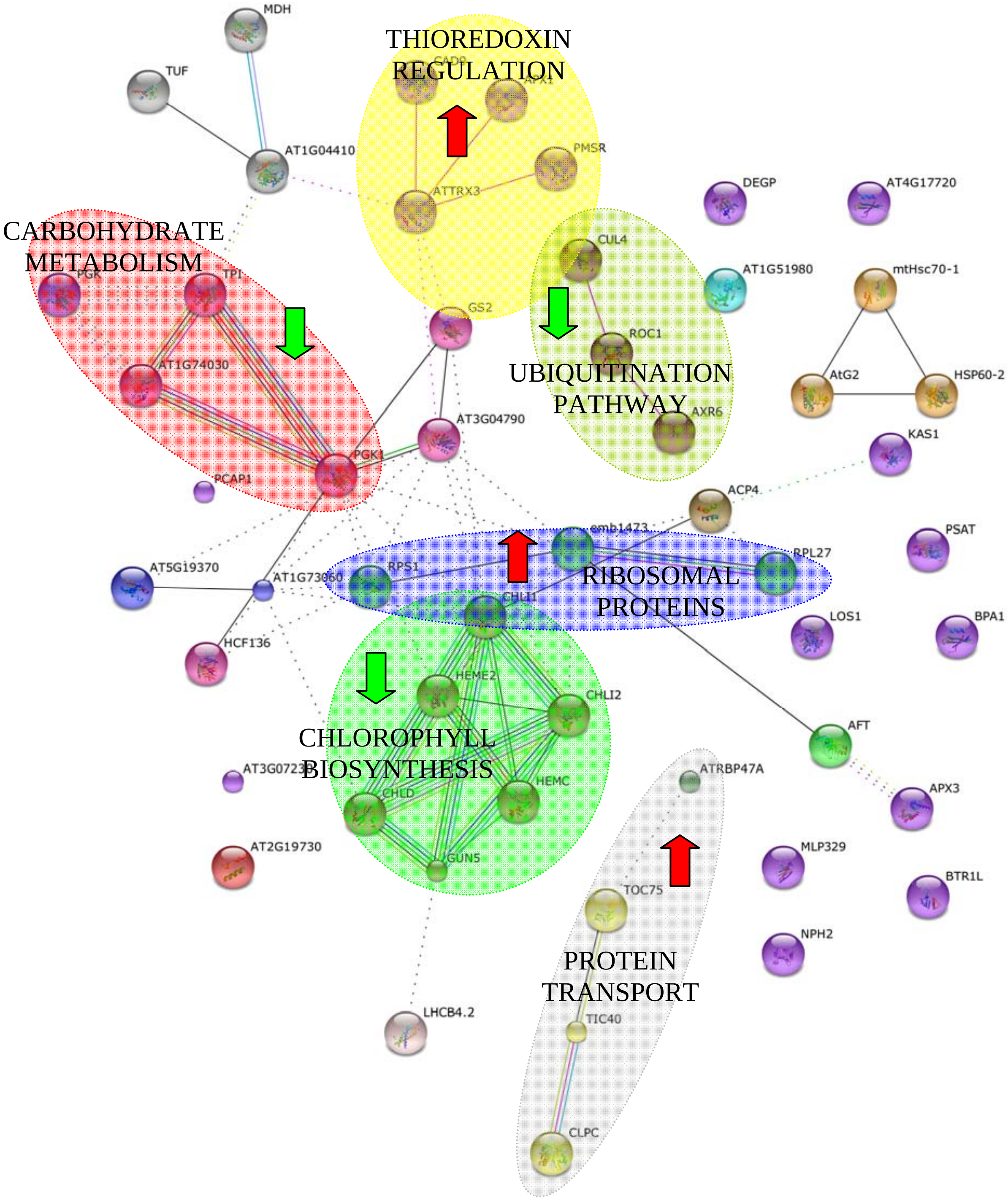

Fig. S8 Protein-protein interaction network constructed using STRING (http://string-db.org; Szklarczyk et al., 2011). String analysis indicates that proteins with similar responses to *HvCKX2* and *ipt* activation are associated with processes including chlorophyll biosynthesis (decreased), carbohydrate metabolism (decreased), thioredoxin regulation (increased), protein transport (increased), proteasome-mediated degradation (decreased), and ribosome biogenesis (increased).

String Input:

AT1G04410	malate dehydrogenase, cytosolic, putative; malate dehydrogenase, cytosolic, putative; FUNCTIONS IN- in 6 functions; INVOLVED IN- response to cadmium i [...]
APX1	APX1 (ascorbate peroxidase 1); L-ascorbate peroxidase; Encodes a cytosolic ascorbate peroxidase APX1. Ascorbate peroxidases are enzymes that scavenge [...]
ATRBP47A	ATRBP47A (Arabidopsis thaliana RNA-binding protein 47a); RNA binding; Arabidopsis thaliana RNA-binding protein 47a (ATRBP47A); FUNCTIONS IN- RNA bindi [...]
AT1G51980	mitochondrial processing peptidase alpha subunit, putative; mitochondrial processing peptidase alpha subunit, putative; FUNCTIONS IN- metalloendopepti [...]
LOS1	LOS1; copper ion binding / translation elongation factor/ translation factor, nucleic acid binding; encodes a translation elongation factor 2-like pro [...]
AT1G73060	unknown protein; unknown protein; FUNCTIONS IN- molecular_function unknown; INVOLVED IN- biological_process unknown; LOCATED IN- chloroplast stroma, c [...]
AT1G74030	enolase, putative; enolase, putative; FUNCTIONS IN- phosphopyruvate hydratase activity; INVOLVED IN- in 12 processes; LOCATED IN- phosphopyruvate hydr [...]
emb1473	emb1473 (embryo defective 1473); structural constituent of ribosome; embryo defective 1473 (emb1473); FUNCTIONS IN- structural constituent of ribosome [...]
MLP329	MLP329 (MLP-LIKE PROTEIN 329); copper ion binding; MLP-LIKE PROTEIN 329 (MLP329); FUNCTIONS IN- copper ion binding; INVOLVED IN- response to biotic st [...]
AT2G19730	60S ribosomal protein L28 (RPL28A); 60S ribosomal protein L28 (RPL28A); FUNCTIONS IN- structural constituent of ribosome; INVOLVED IN- translation, ri [...]
HSP60-2	HSP60-2 (HEAT SHOCK PROTEIN 60-2); ATP binding; HEAT SHOCK PROTEIN 60-2 (HSP60-2); FUNCTIONS IN- ATP binding; INVOLVED IN- response to salt stress, in [...]
HEME2	HEME2; uroporphyrinogen decarboxylase; HEME2; FUNCTIONS IN- uroporphyrinogen decarboxylase activity; INVOLVED IN- porphyrin biosynthetic process; LOCA [...]
AT3G04790	ribose 5-phosphate isomerase-related; ribose 5-phosphate isomerase-related; FUNCTIONS IN- ribose-5-phosphate isomerase activity; INVOLVED IN- defense [...]
AT3G07230	wound-responsive protein-related; wound-responsive protein-related; FUNCTIONS IN- molecular_function unknown; INVOLVED IN- biological_process unknown; [...]
LHCB4.2	LHCB4.2 (light harvesting complex PSII); chlorophyll binding; Lhcb4.2 protein (Lhcb4.2, protein involved in the light harvesting complex of photosyste [...]
DEGP	DEGP1 (DegP protease 1); serine-type endopeptidase/ serine-type peptidase; Encodes a DegP protease; nuclear gene encoding chloroplast-targeted proteas [...]
TOC75	TOC75-III (TRANSLOCON AT THE OUTER ENVELOPE MEMBRANE OF CHLOROPLASTS 75-III); P-P-bond-hydrolysis-driven protein transmembrane transporter; Component [...]
MDH	MDH (MALATE DEHYDROGENASE); L-malate dehydrogenase/ binding / catalytic/ malate dehydrogenase/ oxidoreductase/ oxidoreductase, acting on the CH-OH gro [...]
AtG2	ATG2; aminopeptidase/ metalloexopeptidase; putative nuclear DNA-binding protein G2p (AtG2) mRNA, (401 aa) [...]
TPI	TPI (TRIOSEPHOSPHATE ISOMERASE); triose-phosphate isomerase; Encodes triosephosphate isomerase. (254 aa) [...]
TUF	TUF (VACUOLAR ATP SYNTHASE SUBUNIT E1); proton-transporting ATPase, rotational mechanism; Encodes a vacuolar H+-ATPase subunit E isoform 1 which is re [...]
AT4G17720	RNA recognition motif (RRM)-containing protein; RNA recognition motif (RRM)-containing protein; FUNCTIONS IN- oxidoreductase activity, nucleotide bind [...]
PCAP1	DREPP plasma membrane polypeptide family protein; Encodes a Ca2+ and Cu2+ binding protein. N-terminal myristylation on glycine 2 appears to enable it [...]
ACP4	ACP4 (acyl carrier protein 4); acyl carrier; encodes an acyl carrier protein predominantly expressed in leaves. Gene expression is upregulated by ligh [...]
PMSR	peptide methionine sulfoxide reductase, putative; peptide methionine sulfoxide reductase, putative; FUNCTIONS IN- peptide-methionine-(S)-S-oxide reduc [...]
APX3	APX3 (ASCORBATE PEROXIDASE 3); L-ascorbate peroxidase; Encodes a microsomal ascorbate peroxidase APX3. Ascorbate peroxidases are enzymes that scavenge [...]
PSAT	PSAT; O-phospho-L-serine-2-oxoglutarate aminotransferase; Encodes a phosphoserine aminotransferase which is involved in serine biosynthesis in the chl [...]
mtHsc70-1	mtHsc70-1 (mitochondrial heat shock protein 70-1); ATP binding; mitochondrial heat shock protein 70-1 (mtHsc70-1); FUNCTIONS IN- ATP binding; INVOLVED [...]
ROC1	ROC1 (ROTAMASE CYP 1); peptidyl-prolyl cis-trans isomerase; Encodes cytosolic cyclophilin ROC1. ; PPIases accelerate the folding of proteins. It catal [...]
CAD9	CAD9 (CINNAMYL ALCOHOL DEHYDROGENASE 9); binding / catalytic/ oxidoreductase/ zinc ion binding; CINNAMYL ALCOHOL DEHYDROGENASE 9 (CAD9); FUNCTIONS IN- [...]
BTR1L	BTR1L (BINDING TO TOMV RNA 1L (LONG FORM)); nucleic acid binding; Gene model AT5G04430.1 produces active protein. (BTS1S). Binds to ToMV genomic RNA a [...]

BPA1	BPA1 (BINDINGPARTNEROFACD11 1); nucleic acid binding / nucleotide binding / oxidoreductase; BINDINGPARTNEROFACD11 1 (BPA1); FUNCTIONS IN- oxidoreducta [...]
AT5G19370	rhodanese-like domain-containing protein / PPIC-type PPIASE domain-containing protein; rhodanese-like domain-containing protein / PPIC-type PPIASE dom [...]
HCF136	HCF136; protein binding; encodes a stability and/or assembly factor of photosystem II ; Essential for photosystem II (PSII) biogenesis; required for a [...]
RPS1	RPS1 (RIBOSOMAL PROTEIN S1); RNA binding / structural constituent of ribosome; RIBOSOMAL PROTEIN S1 (RPS1); FUNCTIONS IN- structural constituent of ri [...]
GS2	GS2 (GLUTAMINE SYNTHETASE 2); glutamate-ammonia ligase; chloroplastic glutamine synthetase ; The light-modulated chloroplast/mitochondrial enzyme, enc [...]
RPL27	RPL27 (RIBOSOMAL PROTEIN LARGE SUBUNIT 27); structural constituent of ribosome; RIBOSOMAL PROTEIN LARGE SUBUNIT 27 (RPL27); FUNCTIONS IN- structural c [...]
ATTRX3	ATTRX3 (THIOREDOXIN 3); oxidoreductase, acting on sulfur group of donors, disulfide as acceptor; encodes a cytosolic thioredoxin that reduces disulfid [...]
CHLI2	CHLI2 (MAGNESIUM CHELATASE I2); ATPase/ magnesium chelatase; encodes a second Chl I gene (CHLI2), a subunit of magnesium chelatase which is required f [...]
KAS1	KAS I (3-KETOACYL-ACYL CARRIER PROTEIN SYNTHASE I); catalytic/ fatty-acid synthase; 3-ketoacyl-acyl carrier protein synthase I (KAS I) mRNA, ; Catalyz [...]
CLPC	CLPC1; ATP binding / ATP-dependent peptidase/ ATPase; Encodes a protein that is similar to ATP-dependent Clp protease ATP-binding subunit / ClpC. Invo [...]
NPH2	PHOT2 (PHOTOTROPIN 2); FMN binding / blue light photoreceptor/ kinase/ protein serine/threonine kinase; Membrane-bound protein serine/threonine kinase [...]

Predicted Functional Partners:

GUN5	GUN5 (GENOMES UNCOUPLED 5); magnesium chelatase; Encodes magnesium chelatase involved in plasti [...] (1381 aa)
AXR6	ATCUL1 (ARABIDOPSIS THALIANA CULLIN 1); protein binding; Encodes a cullin that is a component o [...] (738 aa)
TIC40	TIC40; chloroplast protein import (Tic40) (447 aa)
CUL4	CUL4 (CULLIN4); protein binding / ubiquitin-protein ligase; Arabidopsis CULLIN4 (CUL4) forms an [...] (792 aa)
AFT	AKR2 (ANKYRIN REPEAT-CONTAINING PROTEIN 2); protein binding; Involved in targeting of chloropla [...] (342 aa)
PGK	PGK (PHOSPHOGLYCERATE KINASE); phosphoglycerate kinase; Encodes cytosolic phosphoglycerate kina [...] (401 aa)
CHLD	CHLD; ATP binding / magnesium chelatase/ nucleoside-triphosphatase/ nucleotide binding; CHLD; F [...] (760 aa)
PGK1	PGK1 (PHOSPHOGLYCERATE KINASE 1); phosphoglycerate kinase; nuclear phosphoglycerate kinase (PGK [...] (481 aa)
HEMC	HEMC (HYDROXYMETHYLBILANE SYNTHASE); hydroxymethylbilane synthase; Encodes a protein with porph [...] (382 aa)
CHLI1	CHLI1; ATPase/ magnesium chelatase; Encodes the CHLI subunit of magnesium chelatase which is re [...] (424 aa)

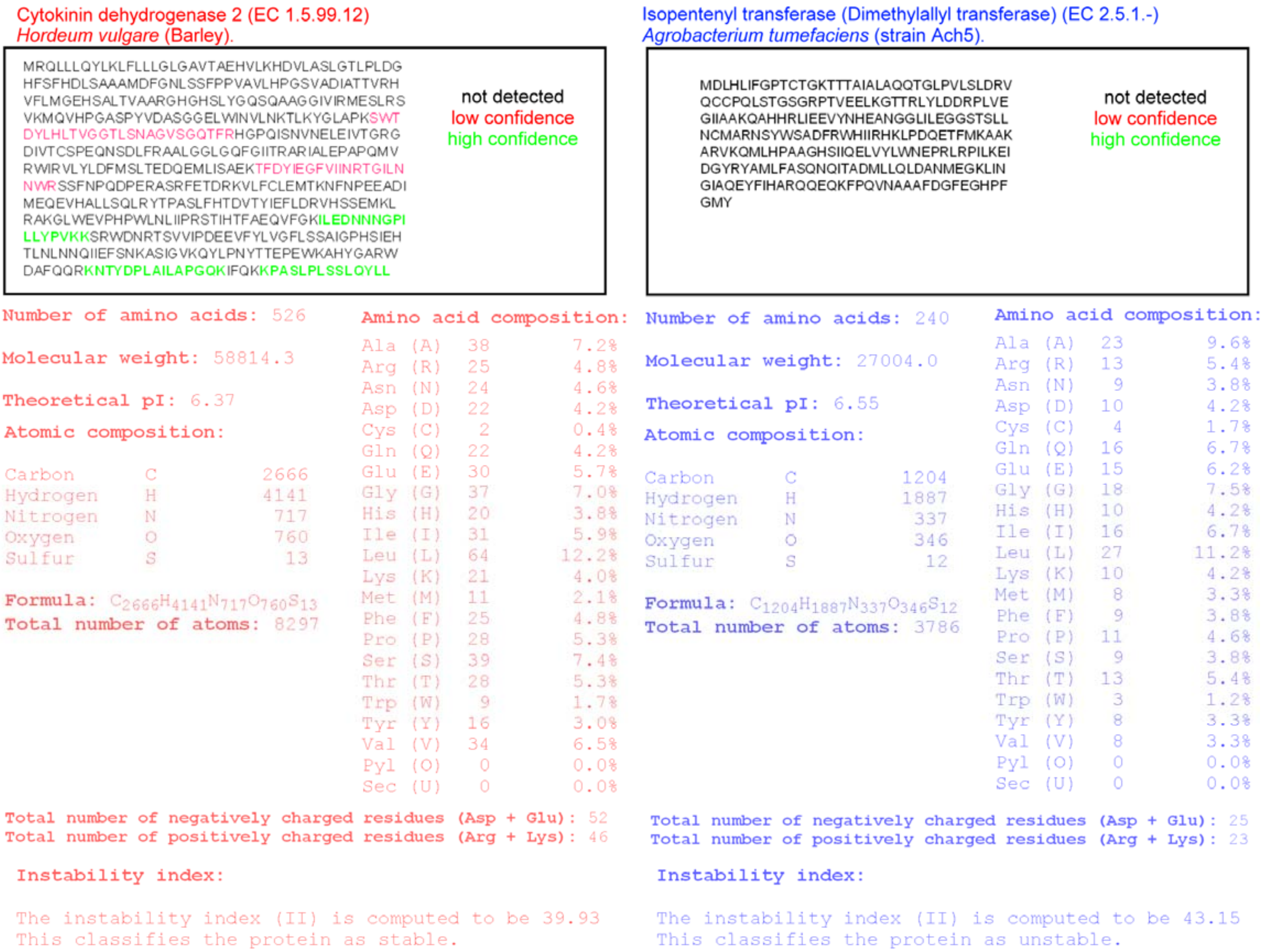

Fig. S9 Expression of *ipt* is evident, but not detectable on the protein level

Following *HvCKX2* activation, HvCKX2 was readily detectable in the LC-MS experiment (Table S8) and its activity is evident from dynamics of the CK pool. However, following *ipt* activation, the ipt level remained below the LC-MS detection limit. The high effectiveness of DEX induction in the *ipt* line was shown on transcript and cytokinin pool levels by Hradilová *et al*. (2007) and Kuderová *et al*. (2008), and ipt activity is reflected in depletion of the isopentenyl precursor 3-hydroxy-3-methylglutarate reported here. Well-known cytokinin-induced polyamine biosynthesis (Walker *et al.*, 1988) is also evident in the metabolome profiles obtained following *ipt* activation. Thus, ipt apparently has low stability. Indeed, ProtParam calculated the ipt instability index at 43.15, which classifies the protein as unstable (Guruprasad *et al.*, 1990).

References:

Guruprasad K, Reddy BV, Pandit MW. 1990. Correlation between stability of a protein and its dipeptide composition: a novel approach for predicting in vivo stability of a protein from its primary sequence. *Protein Engineering* **4**, 155-161.

Hradilová J, Malbeck J, Brzobohatý B. 2007. Cytokinin regulation of gene expression in the AHP gene family in *Arabidopsis thaliana*. *Journal of Plant Growth*

Kuderová A, Urbánková I, Válková M, Malbeck J, Brzobohatý B, Némethová D, Hejátko J. 2008. Effects of conditional IPT-dependent cytokinin overproduction on root architecture of Arabidopsis seedlings. *Plant and Cell Physiology* **49**, 570-582.

Walker MA, Roberts DR, Dumbroff EB. 1988. Effects of cytokinin and light on polyamines during the greening response of cucumber cotyledons. *Plant and Cell Physiology* **29**, 201-205.
