# Supplementary material for: Proteome and metabolome profiling of cytokinin action in Arabidopsis identifying both distinct and similar responses to cytokinin down- and up-regulation
Source: J Exp Bot. 2013 Sep 24;64(14):4193–206. doi: 10.1093/jxb/ert227 (PMC3808309; doi:10.1093/jxb/ert227)
Supplement: Supplementary Data [file supp_ert227_jexbot098541_file003.pdf]

Proteome and metabolome profiling of cytokinin action in Arabidopsis identifying both distinct and similar responses to cytokinin down- and up-regulation

Martin Černý, Alena Kuklová, Wolfgang Hoehenwarter, Lena Fragner, Ondřej Novák, Gabriela Rotková, Petr L. Jedelský, Kateřina Žáková, Mária Šmehilová, Miroslav Strnad, Wolfram Weckwerth, Břetislav Brzobohatý

Methods S1

Overview of the experiment

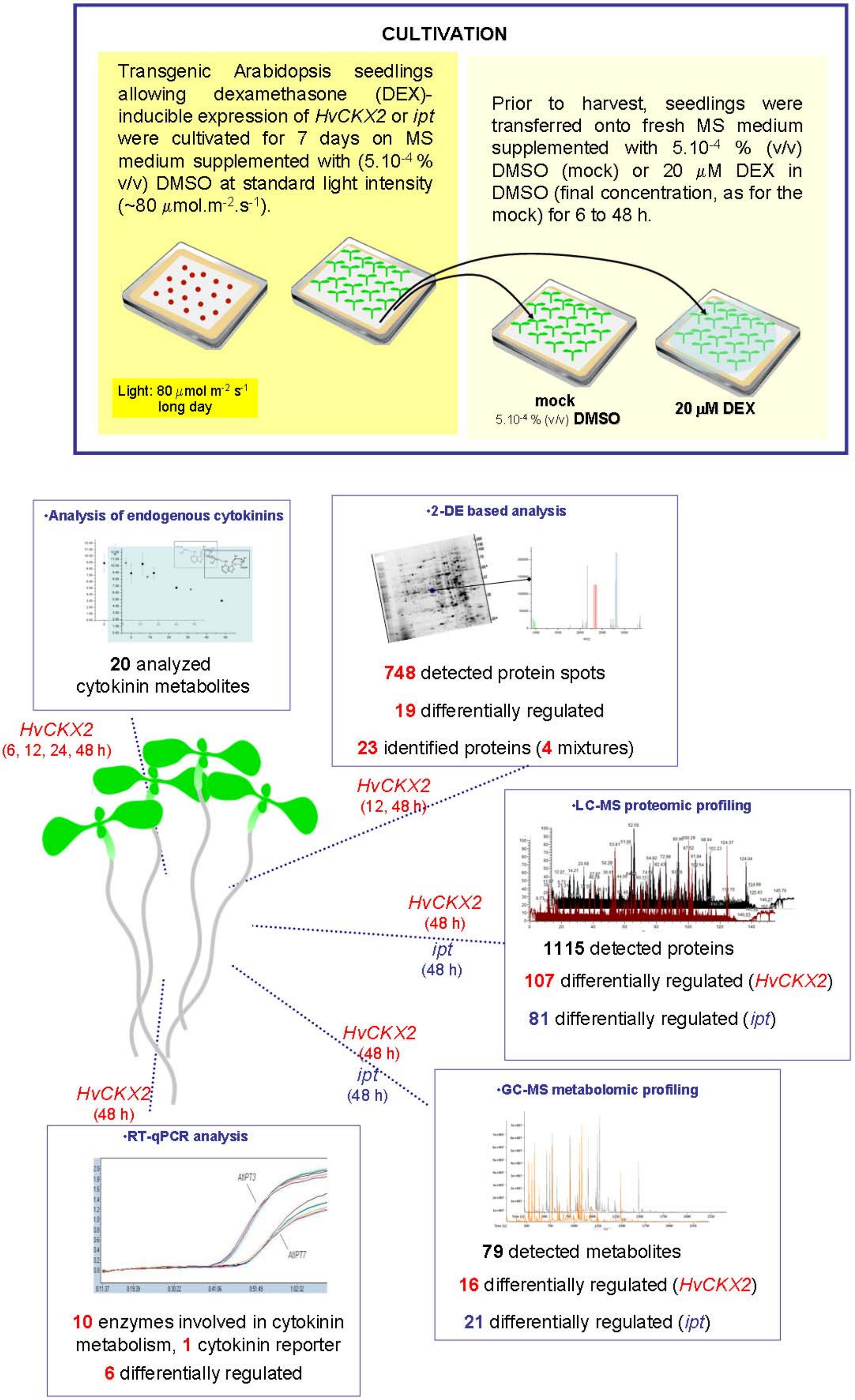

2-DE based proteome analysis

(1) RuBisCO depletion

Protein was extracted from frozen seedlings (180 mg, approximately 250 seedlings) in 0.5 ml TBS buffer (10 mM Tris-HCl, 150 mM NaCl, pH 7.4) supplemented with protease inhibitors and PVPP (Sigma-Aldrich), clarified by centrifugation, loaded onto a Seppro IgY-Rubisco Spin Column (GenWay Biotech) and processed according to the supplier's manual. RuBisCO-depleted samples were pooled, concentrated using an Amicon Ultra-15 Centrifugal Filter Unit and extracted by acetone/TCA extraction (Damerval *et al.*, 1986). Dried protein was solubilized in SOL buffer: 7 M urea, 2 M thiourea, 2% (w/v) CHAPS, 90 mM DTT and the protein concentration was determined (Bradford, 1976) (Sigma-Aldrich).

(2) 2-DE analysis

Solubilized protein was then diluted 1:1 with rehydration solution [SOL supplemented with 1% (v/v) ampholytes pH 3-10, 0.2% (w/v) bromophenol blue] and loaded onto 3-10NL IPG strips (Bio-Rad). Proteins were separated essentially as previously described (Lochmanová *et al.*, 2008). Briefly, portions containing 150 μg of protein were applied to 7 cm IPG strips with a non-linear pH gradient (3-10). The strips were rehydrated for 16 h at room temperature (passive rehydration), then the proteins were isoelectrically focused at 22 °C in six steps in a PROTEAN IEF Cell unit (Bio-Rad): 150 V (20 min), 300 V (20 min), 600 V (20 min), 1500 V (20 min), 3000 V (20 min) and 4000 V up to 12 000 Vh. The strips were then treated with buffers containing DTT and iodoacetamide (Sigma-Aldrich) to reduce and alkylate the proteins, which were then separated by 8-20% linear gradient polyacrylamide concentration SDS–PAGE with the following settings: 100 V (10 min) followed by 150 V (50 min), using a Mini-PROTEAN 3 Dodeca Cell (Bio-Rad). Gels were stained with colloidal Bio-Safe Coomassie G-250 (Bio-Rad) and scanned with a Bio-Rad GS-800 Calibrated Densitometer (700 dpi). Acquired images were analyzed using Decodon Delta 2D software (http://www.decodon.com). Two biological replicates with three technical replicates were used in the comparisons. Responses to DEX activation of proteins corresponding to detected spots were deemed significant if there was a DEX/mock spot volume ratio ≥ ±1.4, with p-values ≥0.05 and similar profiles in two biological replicates. Only spots with significant and reproducible changes were considered for MS identification.

(3) MALDI TOF/TOF protein identification

Proteins were identified as previously described (Hradilová *et al.*, 2010) with minor modifications. Briefly, selected protein spots were digested with trypsin. The dried tryptic peptides were each dissolved in 10 μl of 0.1% trifluoroacetic acid and purified using ZipTip C18 tips and eluted directly on sample **plates** with 10 mg ml^{-1} CHCA in 50% v/v acetonitrile and 0.1% trifluoroacetic acid. Spectra were acquired using **a** 4800 Plus MALDI TOF/TOF analyzer (AB Sciex) equipped with a Nd:YAG laser (355 nm) with firing rate 200 Hz. All spots were measured in MS mode and then up to 10 of the strongest precursors were selected for MS/MS which was performed with 1 kV collision energy and collision cell operating pressure set to 10^{-6} Torr. MS and MS/MS spectra were searched by local Mascot v. 2.1 (Matrix Science) against the TAIR9 database of Arabidopsis protein sequences (33410 sequences). Database search criteria were as follows: enzyme – trypsin; taxonomy – *Arabidopsis thaliana*; fixed modification – carbamidomethylation; variable modification - methionine oxidation; peptide tolerance - 80 ppm, one missed cleavage allowed; MS/MS tolerance - 0.2 Da.

Quantification and identification of endogenous cytokinins

Endogenous cytokinin contents of duplicate samples were analyzed using the method described by Novák *et al.* (2003), with modifications described by Novák *et al.* (2008). The following endogenous cytokinins were analyzed:

tZ	*trans*-ZEATIN
cZ	*cis*-ZEATIN
iP	N^6-ISOPENTENYLADENINE
tZR	*trans*-ZEATIN RIBOSIDE
cZR	*cis*-ZEATIN RIBOSIDE
DHZR	DIHYDROZEATIN RIBOSIDE
iPR	N^6-ISOPENTENYLADENOSINE
tZOG	*trans*-ZEATIN-O-GLUCOSIDE
cZOG	*cis*-ZEATIN-O-GLUCOSIDE
DHZOG	DIHYDROZEATIN-O-GLUCOSIDE
tZROG	*trans*-ZEATIN-O-GLUCOSIDE RIBOSIDE
DHZROG	DIHYDROZEATIN-O-GLUCOSIDE RIBOSIDE
tZ7G	*trans*-ZEATIN-7-GLUCOSIDE
DHZ7G	DIHYDROZEATIN-7-GLUCOSIDE
iP7G	N^6-ISOPENTENYLADENINE-7-GLUCOSIDE
tZ9G	*trans*-ZEATIN-9-GLUCOSIDE
cZ9G	*cis*-ZEATIN-9-GLUCOSIDE
iP9G	N^6-ISOPENTENYLADENINE-9-GLUCOSIDE
tZR5'MP	*trans*-ZEATIN RIBOSIDE-5’-MONOPHOSPHATE
iPR5'MP	N^6-ISOPENTENYLADENOSINE-5’-MONOPHOSPHATE

LC-MS based proteome profiling

(1) Isolation

Proteomic analyses were performed using a gel-free shotgun protocol based on nano-HPLC and MS/MS, as described elsewhere, e.g. Larrainzar *et al.*, 2007). Two biological replicates, each consisting of approximately 300 Arabidopsis seedlings cultivated as described above, were pooled and analyzed in three technical replicates. Frozen seedlings (450 mg, approximately 600 seedlings) were homogenized in an MM 400 mill (Retsch). The homogenized tissue was washed with 1.5 ml acetone (4 °C, 30 min), clarified by centrifugation, washed with 10% (w/v) TCA in acetone, 10% (w/v) TCA in distilled water then 80% (v/v) acetone, resuspended in 0.8 ml SDS buffer [2% (w/v) SDS, 30% (w/v) sucrose, 5% (v/v) β-mecraptoethanol, 5 mM EDTA, 100 mM Tris, pH 8.0], and protein was extracted by 0.4 ml buffer-saturated phenol. Phenolic phase was collected and protein was precipitated overnight in 1.6 ml ice-cold 100 mM ammonium acetate in methanol (-20 °C). Protein pellets were washed with 1.0 ml 80% (v/v) acetone in distilled water, dried and dissolved in 100 mM NH_4HCO_3, 8 M urea. The protein concentration was estimated by the Bradford assay (Bradford, 1976) (Sigma-Aldrich), samples were diluted 1:1 with 20% (v/v) acetonitrile in 100 mM NH_4HCO_3, subjected to in-solution digestion with endoproteinase Lys-C (1 μg per 500 μg of protein, 12 h, 30 °C)(Promega), then further diluted 1:1 with 10% (v/v) acetonitrile in 25 mM NH_4HCO_3, and digested with immobilized trypsin beads (Promega) at 37 °C overnight.

(2) Measurements

The resulting peptides were desalted (SPEC plate C18, Agilent), dried and dissolved in 0.5% (v/v) formic acid in 5% (v/v) acetonitrile, and analyzed online by nanoflow C18 reverse-phase liquid chromatography, loading 5 μg of protein onto a 15 cm Ascentis Express Column (0.1 mm inner diameter; Sigma-Aldrich) and an Eksigent UPLC system (Eksigent) directly coupled to an ESI source and an LTQ-Orbitrap XL mass spectrometer (Thermo Scientific). Peptides were eluted with a linear 155-min 5% to 95% acetonitrile gradient. The mass spectrometer was operated in positive ion mode using data-dependent automatic switching between MS and MS/MS acquisition modes. Fourier-transformed full scan mass spectra were acquired at a target value of 9E05 ions with resolution r = 30 000 at m/z 400 and an m/z range of 300–2000. The seven most intense ions were selected for collision-induced dissociation with a target value of 5000 ions in the LTQ. After MS analysis, raw files were searched against the TAIR10 Arabidopsis database using the Sequest algorithm. For identification and spectral count-based data matrix generation Proteome Discoverer (v 1.3, Thermo Scientific) was used. Database search criteria were as follows: TAIR10 database; enzyme – trypsin, two missed cleavages allowed; variable modification – acetylation (N-terminus), methionine oxidation, phosphorylation (S, T, Y), max. four modifications per peptide; peptide tolerance - 7 ppm, MS/MS tolerance - 0.4 Da; decoy database search - target FDR 0.01. Only high confidence peptides (false discovery rate < 1%) with better than 7 ppm precursor mass accuracy and at least one distinct peptide per protein met identification criteria.

(3) Data comparison

Quantitative differences in protein abundance between DEX- and mock-treated samples were determined by spectral counting (Neilson *et al.*, 2011) and further manually validated by comparison of respective peptide peak areas (Qual Browser 2.0.7, Thermo Scientific). Quantitative differences were deemed significant if there was an absolute DEX/mock ratio ≥1.5, with t-test p-values <0.05.

Metabolome analysis

(1) Extraction

Briefly, two biological replicates, each consisting of approximately 100 Arabidopsis seedlings cultivated as described above, were pooled and analyzed in three technical replicates. Frozen seedlings (150 mg, approximately 200 seedlings) were homogenized in an MM 400 mill (Retsch). Metabolites were extracted with 1 ml methanol/chloroform/distilled water buffer (2.5:1:0.5 [v/v/v]), 4 °C, 8 min; and clarified by centrifugation. The resulting polar phase was separated by adding 0.5 ml of distilled water, divided into two equal parts, dried in a speed-vac concentrator (Thermo-Scientific), and stored at -80 °C until GC-MS analysis.

(2) Derivatisation and GC-MS measurement

Samples were dissolved in 20 µl methoxyamine hydrochloride in dry pyridine (40 mg/ml) and incubated for 90 min at 30 °C with rigorous shaking. They were then treated with 80 µl *N*-methyl-*N*-trimethylsilyltrifluoroacetamide spiked with retention time index markers (alkanes C10-C40, 60 µl/1 ml; Sigma-Aldrich), and incubated for an additional 30 min at 37 °C. They were then analyzed by GC-TOF-MS using an Agilent 6890 gas chromatograph (Agilent, Böblingen, Germany) coupled to a Pegasus IV TOF mass spectrometer (LECO Corp Inc., St. Joseph, MI, USA). One microliter of sample was injected in split (1:10) mode. For GC separation a HP5-MS capillary column (30 m, 0.25 mm I.D., 25 µm film; Agilent) was used with a 40 min temperature gradient (70 °C for 1 min followed by 9 °C per min gradient to 350 °C). The MS acquisition rate was 20 scans/s in the mass range $m/z = 40–600$.

(3) Data analysis

The acquired data were analyzed using ChromaTOF software (LECO), which enables automated data processing. Spectra were calibrated to the retention time index markers, the annotations of selected spectra of mock- and DEX-treated samples were manually checked and a reference peak table with ion traces specific for each analyte to be quantified was created. All chromatograms were compared to the reference and the peak areas were calculated. Quantitative differences in metabolite abundance were deemed significant if there was an absolute DEX/mock ratio ≥1.5, with t-test p-values <0.05.

RT-qPCR analysis

(1) RNA isolation and reverse transcription

RNA was prepared from 50 mg seedlings that had been frozen in liquid nitrogen, using TRIzol reagent (Invitrogen) according to the manufacturer's instructions. To remove contaminating DNA, 1 U of DNase I (Top-Bio, Czech Republic) per 1 μg RNA was used. The resulting samples were incubated for 45 min at 37 ºC, then denatured at 65 ºC for 15 min. First-strand cDNA was prepared using SuperScript II reverse transcriptase (Invitrogen) and oligo(dT) primer according to the manufacturer's instructions. cDNA synthesis was performed with 4 μg of total RNA.

(2) Quantitative PCR

qPCR with specific UPL probes (Roche) and primers designed by ProbeFinder Software was performed using a LightCycler® 480 Instrument and LightCycler® 480 Probes Master (both Roche). Three independent biological replicates and three technical replicates were included for each PCR amplification. Expression levels were normalized to four reference genes and fold-changes in transcript levels were calculated using the ΔΔCT Method with verification of similar efficiencies (Livak and Schmittgen, 2001). Presented results are means obtained for biological replicates and corresponding standard deviations. The significance of differences between activated and non-activated plants was evaluated by Student's t-test ($p<0.05$). For details about primer sequences see supplementary tables.

(3) qPCR conditions

PCR MIX

Component	Final Conc.
Forward Primer	400 nM
Reverse Primer	400 nM
UPL Probe	200 nM
LightCycler® 480 Probes Master	1x
Water	-
Total Volume	7,5 μl PCR MIX + 2,5 μl cDNA

LightCycler® 480 Instrument programming

Pre-incubation	Amplification
10 min at 95ºC	45 cycles: 10 s at 95ºC<br>30 s at 60ºC<br>1 s at 72ºC (single acquisition)<br>cooling: 30 s at 40ºC

Data analysis

Hierarchical clustering analysis by Gene Cluster 3.0 (http://bonsai.hgc.jp/~mdehoon/software/cluster/) was used to organize profiles of responsive protein spots, proteins, hormones and metabolites. Java TreeView 1.1.4r3 (http://jtreeview.sourceforge.net) was used to view the clustering results generated by Cluster 3.0. Information about protein/metabolite function(s) was collected from **the** UniProt database, UniGene database (http://www.ncbi.nlm.nih.gov/unigene), TAIR database (http://www.arabidopsis.org), iHOP portal (http://www.ihop-net.org/UniPub/iHOP/), **a** conserved domains search (http://www.ncbi.nlm.nih.gov/Structure/index.shtml), **a** homology search (http://blast.ncbi.nlm.nih.gov/Blast.cgi), Kyoto Encyclopedia of Genes and Genomes (http://www.kegg.jp/kegg/), and literature.

References

Bradford MM. 1976. A rapid and sensitive method for the quantitation of microgram quantities of protein utilizing the principle of protein-dye binding. *Analytical Biochemistry* **72**, 248-254.

Damerval C, De Vienne D, Zivy M, Thiellement H. 1986. Technical improvements in two-dimensional electrophoresis increase the level of genetic variation detected in wheat-seedling proteins. *Electrophoresis* **7**, 52-54.

Hradilová J, Rehulka P, Rehulková H, Vrbová M, Griga M, Brzobohatý B. 2010. Comparative analysis of proteomic changes in contrasting flax cultivars upon cadmium exposure. *Electrophoresis*, **31**, 421-431.

Larrainzar E, Wienkoop S, Weckwerth W, Ladrera R, Arrese-Igor C, González EM. 2007. *Medicago truncatula* root nodule proteome analysis reveals differential plant and bacteroid responses to drought stress. *Plant Physiology* **144**, 1495-1507.

Livak KJ, Schmittgen TD. 2001. Analysis of relative gene expression data using real-time quantitative PCR and the 2(-Delta Delta C(T)) method. *Melthods* **25**, 402-408.

Lochmanová G, Zdráhal Z, Konečná H, Koukalová Š, Malbeck J, Souček P, Válková M, Kiran NS, Brzobohatý B. 2008. Cytokinin-induced photomorphogenesis in dark-grown Arabidopsis: a proteomic analysis. *Journal of Experimental Botany* **59**, 3705-3719.

Neilson KA, Ali NA, Muralidharan S, Mirzaei M, Mariani M, Assadourian G, Lee A, van Sluyter SC, Haynes PA. 2011. Less label, more free: approaches in label-free quantitative mass spectrometry. *Proteomics* **11**, 535-553.

Novák O, Hauserová E, Amakorová P, Doležal K, Strnad M. 2008. Cytokinin profiling in plant tissues using ultra-performance liquid chromatography-electrospray tandem mass spectrometry. *Phytochemistry*, **69**, 2214-2224.

Novák O, Tarkowski P, Tarkowská D, Doležal K, Lenobel R, Strnad M. 2003. Quantitative analysis of cytokinins in plants by liquid chromatography-single-quadrupole mass spectrometry. *Analytica Chimica Acta* **480**, 207-218.
